# Generation of an accurate CCSD(T)/CBS data set and assessment of DFT methods for the binding strengths of group I metal–nucleic acid complexes

**DOI:** 10.3389/fchem.2023.1296787

**Published:** 2023-11-20

**Authors:** Briana T. A. Boychuk, Sarah P. Meyer, Stacey D. Wetmore

**Affiliations:** Department of Chemistry and Biochemistry, University of Lethbridge, Lethbridge, AB, Canada

**Keywords:** DNA, RNA, alkali metals, biomolecules, chemical structure, interaction energies, computational chemistry

## Abstract

Accurate information about interactions between group I metals and nucleic acids is required to understand the roles these metals play in basic cellular functions, disease progression, and pharmaceuticals, as well as to aid the design of new energy storage materials and nucleic acid sensors that target metal contaminants, among other applications. From this perspective, this work generates a complete CCSD(T)/CBS data set of the binding energies for 64 complexes involving each group I metal (Li^+^, Na^+^, K^+^, Rb^+^, or Cs^+^) directly coordinated to various sites in each nucleic acid component (A, C, G, T, U, or dimethylphosphate). This data have otherwise been challenging to determine experimentally, with highly accurate information missing for many group I metal–nucleic acid combinations and no data available for the (charged) phosphate moiety. Subsequently, the performance of 61 DFT methods in combination with def2-TZVPP is tested against the newly generated CCSD(T)/CBS reference values. Detailed analysis of the results reveals that functional performance is dependent on the identity of the metal (with increased errors as group I is descended) and nucleic acid binding site (with larger errors for select purine coordination sites). Over all complexes considered, the best methods include the mPW2-PLYP double-hybrid and ωB97M-V RSH functionals (≤1.6% MPE; <1.0 kcal/mol MUE). If more computationally efficient approaches are required, the TPSS and revTPSS local meta-GGA functionals are reasonable alternatives (≤2.0% MPE; <1.0 kcal/mol MUE). Inclusion of counterpoise corrections to account for basis set superposition error only marginally improves the computed binding energies, suggesting that these corrections can be neglected with little loss in accuracy when using larger models that are necessary for describing biosystems and biomaterials. Overall, the most accurate functionals identified in this study will permit future works geared towards uncovering the impact of group I metals on the environment and human biology, designing new ways to selectively sense harmful metals, engineering modern biomaterials, and developing improved computational methods to more broadly study group I metal–nucleic acid interactions.

## Introduction

Metal ions are utilized in various aspects of life. For example, metals are involved in basic biological functions, useful for the design of new energy storage materials, and are integral to the development of pharmaceutical drugs to treat diseases (Buccella et al., 2019; Franz and Metzler-Nolte, 2019; Shi et al., 2020). Group I metals in particular play critical roles and are actively exploited in such applications (Gentner and Mulvey, 2021). For example, Na^+^ and K^+^ are essential cofactors for maintaining the structure and function of nucleic acids, stabilizing cell membranes, and aiding ATP utilization (Auffinger et al., 2016; Tomita et al., 2017). Additionally, Na^+^ is commonly used in body soaps, while K^+^ is used in laundry detergents (Lin et al., 2005; Khan et al., 2022). On the other hand, Li^+^ is used in clean energy initiatives, including the production of lithium-ion batteries (Raabe, 2023), and in drugs to treat bipolar disorders (Machado-Vieira et al., 2009). Rb^+^ and Cs^+^ have been utilized in perovskite materials to produce enhanced solar cells (Hu et al., 2018).

Despite their usefulness in our lives, an overabundance of group I metals can occur in our food sources and drinking water through industrial processes such as mining, waste dumping, and emissions from industrial plants (Gomes et al., 2016). As a result, group I metals can accumulate in the environment and human body, which causes various detrimental effects. For example, a surplus of group I metals can alter the pH of soil and water, which can lead to a physiological disturbance in fish, reduce plant growth, and eliminate biological communities (e.g., zooplankton and *Vibrio fischeri*) (Gomes et al., 2016). In the human body, high levels of Li^+^ can result in cerebellar or renal dysfunction (Schrauzer, 2002). Alternatively, an imbalance of Na^+^ and K^+^ can lead to hypertension, which may cause a stroke or heart disease (Levings and Gunn, 2014). On the other hand, Rb^+^ and Cs^+^ are known to be mildly toxic, having the ability to displace K^+^ and interfere with cellular functions (Zhou et al., 2017). As a specific example, increased levels of Rb^+^ or Cs^+^ in the brain have been correlated with the onset of Parkinson’s disease (Ramos et al., 2016). Since group I metals are involved in different aspects of ecological and human biology, as well as disease progression, it is important to understand how these metals interact with biomolecules (e.g., nucleic acids and proteins).

In addition to answering interesting questions in biology, investigating interactions between group I metals and nucleic acids in particular is key to unlocking many new applications. For example, the development of novel biomaterials holds promise in the energy sector. Specifically, nucleic acids have been explored as a way to enhance lithium–sulfur batteries (Li et al., 2015). Alternatively, there is increasing interest in the design of nucleic acid biosensors to detect toxic metals in the environment or body (Zhou et al., 2017). Indeed, nucleic acids have advantageous metal binding properties, inherently possessing numerous metal binding sites that exhibit varying binding affinities and having the ability to fold into diverse 3D architectures that afford unique metal-binding pockets (e.g., G-quadruplexes and helical junctions) (Zhou et al., 2017). As a result, multiple nucleic acid sensors have been designed to target metal ions. For example, DNAzymes are currently available in the market to selectively sense Pb^2+^ (McKernan, 2014), while an aptamer has been designed to specifically target Cd^2+^ (Liu et al., 2023). Among group I metals, the NaA43 DNAzyme has been shown to target Na^+^ in the presence of other mono-, di-, and trivalent metals (Torabi et al., 2015). However, although nucleic acid sensors have also been designed for K^+^ and Cs^+^ (Lin et al., 2016; Zhou et al., 2017), these solutions are not exclusive for the given metal (Lin et al., 2016; Zhou et al., 2017; Zhao et al., 2022) and/or detailed testing of their function in the presence of a wide range of metals has yet to be done, and no sensors have been made to date for other group I metals. Therefore, more work is necessary to establish functional nucleic acid sensors for group I metals, as well as exploit metal–nucleic acid interactions in other applications such as new materials for energy storage.

As a first step to understanding the roles of metals in nature and designing nucleic acid sensors or novel energy storage materials, among other applications, fundamental information about the structure and binding energies of metal–nucleic acid complexes is required. Although early experimental studies used the kinetic method approach to estimate the gas-phase binding energies between Li^+^, Na^+^, or K^+^ and each canonical DNA/RNA nucleobase (Rodgers and Armentrout, 2016), this technique is unable to yield absolute metal ion binding affinities due to the required use of a reference, with unique references employed for different metals also preventing cross-comparisons between metals and/or nucleic acid components (Davidson and Kebarle, 1976; Cerda and Wesdemiotis, 1996). While the absolute gas-phase binding energies of Li^+^, Na^+^, or K^+^ to A, C, T, or U, and Rb^+^ or Cs^+^ to C were determined using threshold collision-induced dissociation (TCID) (Rodgers and Armentrout, 2000; Yang and Rodgers, 2012; Yang and Rodgers, 2014), limitations exist in these studies at least in part due to the lack of structural information regarding the complexes formed and missing metal–nucleobase combinations. TCID measurements can also lack sensitivity, with Li^+^ having been deemed particularly challenging due to a low mass-to-charge ratio and high velocity that results in ineffective metal trapping (Rodgers and Armentrout, 2007). Furthermore, there is a high risk of nucleobase tautomerization (Yang and Rodgers, 2012; Yang and Rodgers, 2014), which may result in complexes that are not relevant to DNA/RNA present in biosystems or nucleic acid-based applications. Although infrared multiple photon dissociation (IRMPD) spectroscopy has also been used to gain information about the complexes formed between Li^+^, Na^+^, K^+^, Rb^+^, or Cs^+^ bound to A (Rajabi et al., 2010), as well as mono- or dihydrated Li^+^ bound to T or U (Gillis et al., 2009; Burt and Fridgen, 2012), the method necessitates the use of computational methods (B3LYP) to obtain the corresponding structures and binding energies. Thus, atomic level information that unequivocally correlates the structures and binding energies for the complete set of possible group I metal–nucleic acid complexes is still required.

To fill in knowledge gaps and complement previous experimental work on group I metal–nucleic acid interactions, several computational studies have been performed (Burda et al., 1996; Rodgers and Armentrout, 2000; Monajjemi et al., 2003; Zhu et al., 2004; Hashemianzadeh et al., 2008; Gillis et al., 2009; Marino et al., 2010; Rajabi et al., 2010; Burt and Fridgen, 2012; Stasyuk et al., 2020; Boychuk et al., 2021). For example, MP2 has been used to investigate the gas-phase structure and stability of select group 1–nucleobase complexes (Burda et al., 1996; Rodgers and Armentrout, 2000; Monajjemi et al., 2003; Hashemianzadeh et al., 2008). However, all possible complexes for each nucleobase and nucleobase binding sites have not been systematically explored in this capacity. B3LYP has been more recently used to investigate a more complete set of group 1 metal–nucleobase complexes (Monajjemi et al., 2003; Zhu et al., 2004; Gillis et al., 2009; Marino et al., 2010; Rajabi et al., 2010; Burt and Fridgen, 2012), but the reliability of DFT (especially the popular, non-dispersion-corrected B3LYP) for the binding affinities of group I metals to various nucleic acid sites remains ambiguous. Although a computational study has provided CCSD(T) reference binding strengths at the complete basis set limit (CBS) for Li^+^ interactions with A, C, G, T, U, or the phosphate moiety, as well as tested the performance of 54 DFT methods (Boychuk et al., 2021), the remaining group I metal–nucleic acid complexes have yet to be explored. Structural and energetic evaluation of complexes involving the remaining group I metals with a high-level of theory is essential for providing accurate chemical information that is currently missing from experimental data sets. Furthermore, it is necessary to establish reliable functionals for the entire group due to known variations in functional performance across different metals (Chan et al., 2019; Grauffel et al., 2021; Boychuk and Wetmore, 2023).

**FIGURE 1:**
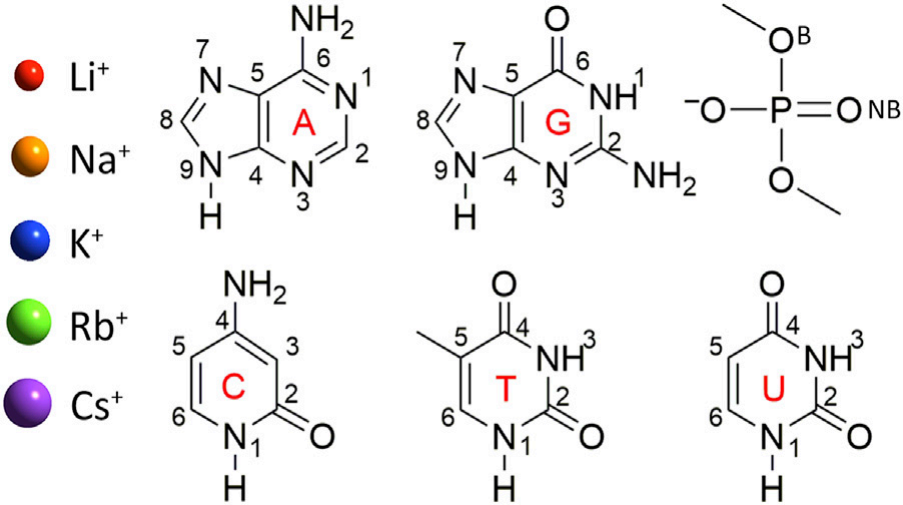
Group I metals and nucleic acid components considered in this study. Structure and chemical numbering provided for nucleic acid components (B, bridging and NB, non-bridging oxygen in the dimethylphosphate model).

To gain fundamental insight into the structures and binding energies of group I metal–nucleic acid complexes as well as identify DFT methods that can accurately describe these interactions for future applications, the present work generates a complete data set of gas-phase binding strengths for group I metal–nucleic acid complexes, which has proven challenging for experimental determination. Specifically, the binding energies of each metal to each canonical DNA/RNA nucleobase (A, C, G, T, and U) and a model for the phosphate moiety (dimethylphosphate; Figure 1) are calculated using CCSD(T) extrapolated to the CBS limit. Subsequently, 61 DFT methods (Table 1) across different functional families according to the metaphorical “Jacob’s Ladder” are tested for their ability to reproduce the CCSD(T)/CBS results. The most reliable and robust DFT methods identified from this work can be applied in future studies to uncover the roles of group I metals in biology, guide the rational design of nucleic acid sensors to target metals in the environment and the human body, and construct new energy storage materials. The highly-accurate data set generated in the present work can also be used to test new functionals and develop new parameters for molecular dynamics (MD) simulations to expand computational studies of group I metal–nucleic acid complexes more broadly.

**TABLE 1:** Functionals tested for their ability to describe group I metal–nucleic acid interactions.

Family	Functional	D3^a^	D4^a^	%HF^b^	%MP2	Ref.
Double-Hybrid	B2-PLYP	X		53	27	Grimme (2006)
	mPW2-PLYP			55	25	Schwabe and Grimme (2006)
	PBE-QIDH			69	33.4	Br et al. (2014)
	PBE0-DH			50	12.5	Brémond and Adamo (2011)
	DSD-PBEP86			68	(51, 23)^d^	Kozuch and Martin (2011), Rodgers and Armentrout, (2016), Sherrill et al. (2017)
RSH	M11			42.8–100^c^		Peverati and Truhlar (2011)
	MN12-SX	X		25–0^c^		Peverati and Truhlar (2012a)
RSH meta-GGA	ωB97M-V			15		Mardirossian and Head-Gordon (2016)
RSH GGA	ωB97					Chai and Head-Gordon (2008a)
	ωB97X	X	X	16		Chai and Head-Gordon (2008a)
	ωB97X-D			22		Chai and Head-Gordon (2008b)
	ωB97X-V			16.7		Mar et al. (2014)
	HSE06	X		25–0^b^		Heyd et al. (2003), Heyd and Scuseria, (2004a), Heyd and Scuseria, (2004b), Heyd et al. (2005), Izmaylov et al. (2006), Krukau et al. (2006), Henderson et al. (2009)
	LC-PBE					Perdew et al. (1996), Iikura et al. (2001)
	LC-ωPBE	X				Vydrov et al. (2006), Vydrov and Scuseria, (2006), Vydrov et al., (2007)
	CAM-B3LYP	X		19–65^b^		Yanai et al. (2004)
GH meta-GGA	M06			27		Zhao and Truhlar (2008)
	M06-2X			54		Zhao and Truhlar (2008)
	M06-HF			100		Zhao and Truhlar (2006a)
	BMK			42		Boese and Martin (2004)
	MN15			44		Yu et al. (2016a)
	PW6B95			28		Zhao and Truhlar (2005)
GH GGA	BH&HLYP			50		Becke (1993a)
	SOGGA11-X			40.15		Peverati et al. (2011)
	B3PW91	X		20		Becke (1993b)
	PBE0	X		25		Adamo and Barone (1999)
	B3LYP	X	X	20		Lee et al. (1988), Becke, (1993b)
	X3LYP	X		21		Xu and Goddard (2004)
	O3LYP	X				Cohen and Handy (2001)
	TPSSh			10		Staroverov et al. (2003), Tao et al. (2003)
	B97-2			21		Wilson et al. (2001)
Local meta-GGA	TPSS	X	X			Tao et al. (2003)
	revTPSS					Perdew et al. (2009a), Perdew et al. (2009b)
	M11-L					Peverati and Truhlar (2012b)
	M06-L					Zhao and Truhlar (2006b)
	MN12-L					Peverati and Truhlar (2012c)
	MN15-L					Yu et al. (2016b)
Local GGA	mPW91					Adamo and Barone (1998)
	BLYP	X				Becke, (1988), Lee et al. (1988), Miehlich et al. (1989)
	BP86	X				Becke, (1988), Lee et al. (1988)
	PBE	X	X			Perdew et al. (1996)
Local LDA	SVWN5					Vosko et al. (1980)

^a^ Functionals indicated by an X were considered with and without the D3(BJ) or D4 empirical dispersion correction.

^b^ Percentage of Hartree-Fock exchange (%HF).

^c^ The first value is the %HF at short range and the second value is the %HF at long range.

^d^ The MP2 component of this functional is calculated using the spin-component-scaled formulism and the two values represent opposite- and same-spin values, respectively.

## Computational methodology

Models were constructed by placing a group I metal (Li^+^, Na^+^, K^+^, Rb^+^, or Cs^+^) at each potential binding site of each nucleobase (A, C, G, T, or U) or the phosphate component (Figure 1). Dimethylphosphate was used to represent the phosphate moiety, which places methyl caps at the location of the connecting sugars to prevent self-interactions between a hydrogen cap and the phosphate moiety, and has been applied in previous work investigating metal–phosphate interactions in the context of nucleic acids (Ruan et al., 2008; Boychuk et al., 2021). Although previous work used MP2/aug-cc-pVTZ to optimize Li^+^–nucleic acid complexes (Boychuk et al., 2021), all metal–nucleic acid complexes investigated in the present work were optimized with MP2(full)/def2-TZVPP for consistency between the metals, with the def2 series being the best basis sets available for heavier group I metals. Frequency calculations were subsequently performed to confirm stable minima. The MP2 geometries were used for all subsequent calculations.

For each metal–nucleic acid complex, the binding energy (BE) was calculated as

$$E^{BE}=E^{dimer}-E^{nucleic\;acid}-E^{metal}$$

where E^*BE*^ is the BE of the complex, E^*dimer*^ is the dimer energy, and E^*nucleic acid*^ and E^*metal*^ are the isolated monomer energies of the nucleic acid subcomponent and metal, respectively. The reference BEs were evaluated at the CCSD(T)/CBS level of theory using the most common approach for obtaining benchmark values for non-covalent interactions (Řezáč and Hobza, 2016). Specifically, the following equation was used

$$E\left(CCSD\left(T\right)/CBS\right)=E\left(HF\right)+E^{corr}\left(MP2/CBS\right)+∆CCSD\left(T\right)$$

where E(HF) was evaluated using def2-QZVPP, E^*corr*^(MP2/CBS) is the MP2 correlation energy extrapolated to the CBS limit using def2-TZVPP and def2-QZVPP according to the Helgaker extrapolation scheme (Helgaker et al., 1997; Halkier et al., 1998), and ΔCCSD(T) was calculated as the difference between the MP2 and CCSD(T) energies evaluated with the def2-TZVPP basis set. The def2 basis sets applied in the present work were previously shown to be well suited for this extrapolation scheme (Neese and Valeev, 2011). All-electron calculations were carried out with CCSD(T) and MP2(full) for the lighter metals (i.e., Li^+^ and Na^+^), while CCSD(T) and MP2 were combined with the Stuttgart Dresden relativistic (RLC) effective core potential (ECP) for the heavier metals (i.e., K^+^, Rb^+^, and Cs^+^). As done in the literature and proposed by Halkier et al. (1999), the averages of the counterpoise uncorrected and corrected binding strengths were evaluated to account for the overestimation and underestimation of true CCSD(T)/CBS BE values, respectively (Kim et al., 1992; Burns et al., 2014; Sherrill et al., 2017; Garcıa et al., 2019; Boychuk et al., 2021).

The accurate CCSD(T)/CBS data set was subsequently used to test the performance of 61 functionals (Table 1) from different families of the hierarchal “Jacob’s Ladder”. These calculations were performed using the same MP2 geometries employed for the CCSD(T)/CBS calculations. We note that previous work has highlighted little impact in functional performance when the geometry of select Li^+^–nucleic acid complexes were re-optimized with DFT (Boychuk et al., 2021). The def2-TZVPP basis set was used throughout, with the Stuttgart Dresden RLC ECP used for the heavier metals (K^+^, Rb^+^, and Cs^+^). We note that previous work on a selection of Li^+^–nucleic acid complexes revealed a negligible difference in DFT functional performance upon basis set expansion to def2-QZVPP (Boychuk et al., 2021). Therefore, due to the large number of functional, metal, and nucleic acid component combinations considered in the present work as well as the intended application of the best methods to larger nucleic acid systems, we employed the computationally more efficient TZ basis set in the present work. The reported binding energies include counterpoise corrections to account for the basis set superposition error (BSSE), which was determined using the Boys and Bernardi scheme, although the impact of neglecting the counterpoise correction is also discussed in the Results and Discussion. For 15 functionals, Grimme’s D3 (Grimme et al., 2010; Grimme et al., 2011) empirical dispersion correction with Becke–Johnson damping (BJ) (Johnson and Becke, 2006) was used, while the D4 (Caldeweyher et al., 2017) model was combined with 4 functionals. All calculations were performed in the gas phase using the default settings in Gaussian 16 (B.01) (Frisch et al., 2016), with the exception of the B3LYP-D4, PBE-D4, TPSS-D4, ωB97X-D4, ωB97X-D3(BJ), ωB97X-V, and ωB97M-V calculations, which were performed using the default settings in ORCA 5.0.2 and the convergence criteria set to “VeryTightSCF” (Neese, 2012).

## Results and discussion

### Generating a highly accurate CCSD(T)/CBS data set of binding strengths for group I metal–nucleic acid complexes

**FIGURE 2:**
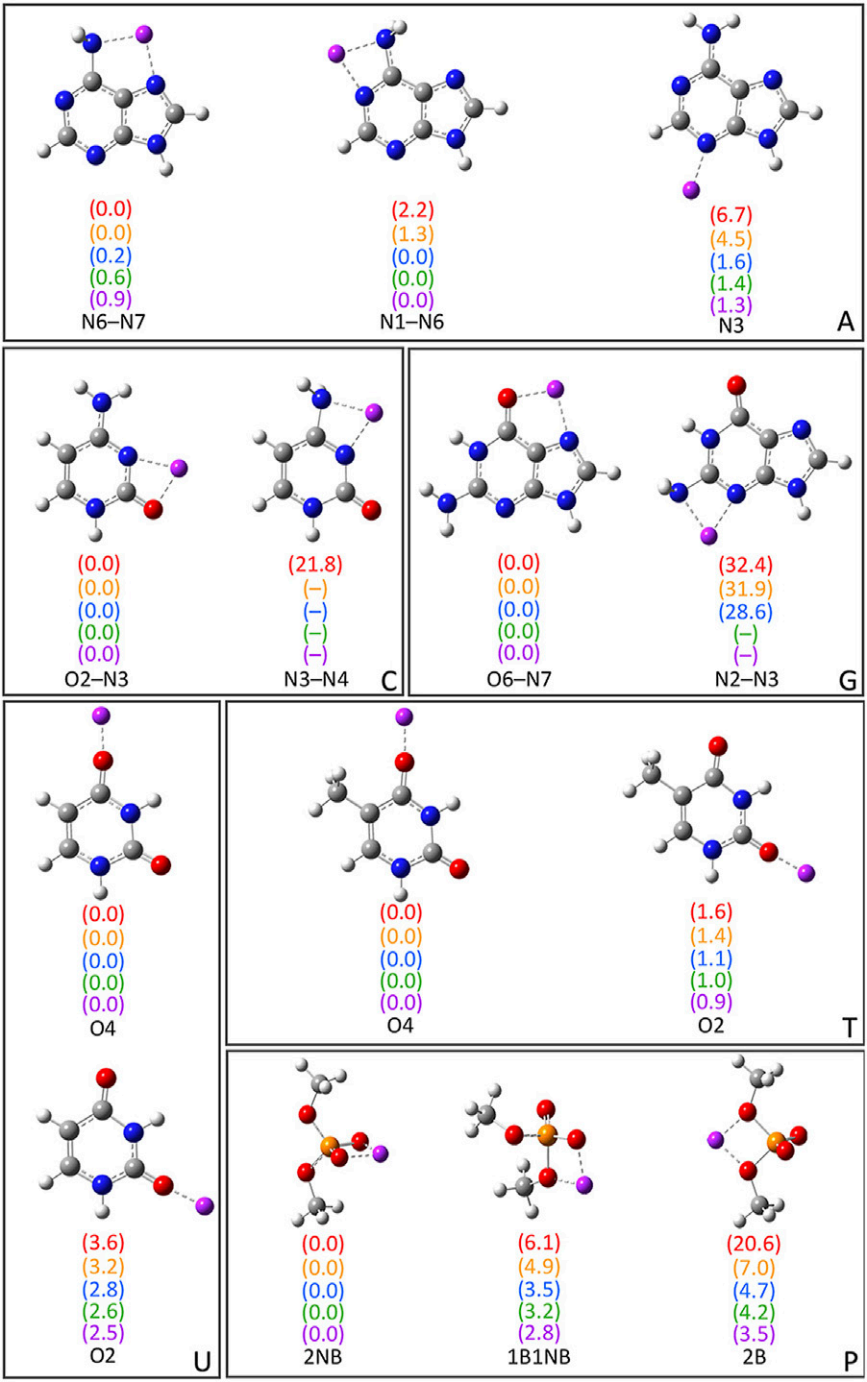
CCSD(T)/CBS//MP2/def2-TZVPP characterized complexes between group I metals and the nucleobases (A, G, C, T, and U) or dimethylphosphate (P). The relative energies for a given metal–nucleic acid component combination (parentheses) are provided in kcal/mol and colored according to metal identity [Li^+^ (red), Na^+^ (orange), K^+^ (blue), Rb^+^ (green), and Cs^+^ (purple)]. The CCSD(T)/CBS binding strengths and MP2/def2-TZVPP coordination distances are provided in Supplementary Tables S1, S2.

As noted in the Computational Methodology, each group I metal (Li^+^, Na^+^, K^+^, Rb^+^, or Cs^+^) was placed at each potential binding site in each nucleic acid component (A, C, G, T, U, or dimethylphosphate; Figure 1). Specifically, for the nucleobases, the N3 and N7 binding sites of the purines were explored, as well as N1 and N6 of A, and O6 of G, while the O2 and O4 binding sites of the pyrimidines were considered, as well as N4 of C. For the nucleic acid backbone, coordination to the bridging (B) and non-bridging (NB) oxygens of dimethylphosphate was considered. 64 unique complexes were identified between a group I metal and a nucleic acid component (Figure 2; Supplementary Table S1). The nucleobase binding sites identified are consistent with structures previously reported in the literature (Burda et al., 1996; Rodgers and Armentrout, 2000; Monajjemi et al., 2003; Zhu et al., 2004; Hashemianzadeh et al., 2008; Gillis et al., 2009; Marino et al., 2010; Rajabi et al., 2010; Burt and Fridgen, 2012; Stasyuk et al., 2020; Boychuk et al., 2021). These structures were used to calculate the corresponding gas-phase CCSD(T)/CBS relative energies for each metal–nucleic acid component combination (Figure 2) and binding strengths for each complex (Supplementary Table S2).

Bidentate complexes were characterized between each group I metal and the N6–N7, and N1–N6 sites of A, which involve rotation of the N6 amino group compared to isolated A. Although the binding strengths are nearly equal at both binding sites for all metals (within ∼2 kcal/mol), Li^+^ and Na^+^ preferentially coordinate to the N6–N7 position (binding energies of −51.1 and −36.0 kcal/mol, respectively), while K^+^, Rb^+^, and Cs^+^ slightly prefer binding at N1–N6 (binding energies of −19.6, −17.1, and −15.1 kcal/mol, respectively). The monodentate complex formed at N3 of A for all metals is ∼4–7 kcal/mol less stable for the lighter group I metals, but within 1.6 kcal/mol for K^+^, Rb^+^, and Cs^+^. For G, the most stable complex for each group I metal involves bidentate coordination at the O6–N7 position, with the binding strength decreasing as Li^+^ (−73.0 kcal/mol) >> Na^+^ >> K^+^ > Rb^+^ > Cs^+^ (−33.4 kcal/mol). Although this is the only G complex characterized for Rb^+^ and Cs^+^, Li^+^, Na^+^, and K^+^ also coordinate to N3 of G, which results in a significantly less stable complex (by ∼29–32 kcal/mol).

For C, the most energetically favorable complex for all group I metals involves bidentate coordination at the O2–N3 position, with the binding strength decreasing as Li^+^ (−69.3 kcal/mol) >> Na^+^ >> K^+^ > Rb^+^ > Cs^+^ (−30.5 kcal/mol). Only Li^+^ forms an additional complex at N3–N4 that is afforded by N4 amino group rotation compared to isolated C, which is 21.8 kcal/mol less stable. Within T and U binding sites, group I metals form monodentate complexes at O4 and O2, with the O4 site being preferred for both nucleobases by up to 1.6 kcal/mol for T and 3.6 kcal/mol for U. In general, there are minimal differences (0.1–0.3 kcal/mol) between the binding strengths at O4 of T and U regardless of metal identity, while the differences range from ∼1.0 to 2.0 kcal/mol at O2.

Three complexes were isolated for each group I metal that involve coordination to dimethylphosphate (P), including those with coordination to one bridging and one non-bridging oxygen (1B1NB), two bridging oxygens (2B), or two non-bridging oxygens (2NB). All metals except Li^+^ also coordinate to one non-bridging oxygen in the P(2B) complex (Supplementary Figure S1). However, the strongest binding interactions for all metals occur at the 2NB position, followed by 1B1NB and then 2B. Nevertheless, the difference in the binding strength as a function of dimethylphosphate site decreases from ∼20 to ∼4 kcal/mol as group I is descended.

Overall, the magnitude of group I metal–nucleic acid interaction energies decreases with an increase in the size of the metal (Li^+^ >> Na^+^ >> K^+^ > Rb^+^ > Cs^+^), which matches the general trends seen in previous experimental (Rodgers and Armentrout, 2016) and computational (Burda et al., 1996; Monajjemi et al., 2003; Zhu et al., 2004; Hashemianzadeh et al., 2008; Marino et al., 2010) studies. Indeed, the metal–ligand distances increase with group I metal size (Supplementary Table S1), which weakens ion–dipole and ion–induced dipole interactions between the metal and nucleic acid component. As a result, there is less distinction between the strongest and weakest binding sites for a given nucleobase as the size of the metal increases, especially for the largest metal Cs^+^. When the preferred binding site for each nucleobase is considered, the group I metal–nucleobase interaction energies decrease as G > C >> A ∼ T = U, which matches the trends predicted by TCID for A, C, T, and U (Rodgers and Armentrout, 2016). Furthermore, the group 1 metal binding affinity to dimethylphosphate is ∼2 (Li^+^ and Na^+^) or ∼3 (K^+^, Rb^+^, and Cs^+^) times greater than that to the most favorable nucleobase site (G(O6–N7)), which correlates with experimental data for trimethylphosphate (Ruan et al., 2008).

In addition to reproducing trends in binding strengths, our CCSD(T)/CBS data are sometimes consistent with TCID predicted interaction energies. For example, the differences between the experimental (Rodgers and Armentrout, 2000) and CCSD(T)/CBS results for T and U are <1 kcal/mol. Nevertheless, deviations exist between the experimental and computational data, which may arise at least in part due to limitations in the experimental methodology. For example, there is a significant difference (2.9 kcal/mol) between our CCSD(T)/CBS and previously published TCID data (Rodgers and Armentrout, 2000) for the Li^+^–A complex, with TCID having a known low sensitivity for Li^+^ (Rodgers and Armentrout, 2007). There is an even larger difference (13 kcal/mol) between the TCID and CCSD(T)/CBS results for Li^+^ bound to C, which may arise from the low Li^+^ sensitivity coupled with nucleobase tautomerization due to the use of thermal vaporization (Yang and Rodgers, 2012; Yang and Rodgers, 2014). Indeed, the experimental (Rodgers and Armentrout, 2000; Yang and Rodgers, 2014) and theoretical results for the remaining group I metals (Na^+^, K^+^, Rb^+^, and Cs^+^) bound to C or A deviate by ∼1–4 kcal/mol, possibly due at least in part to nucleobase tautomerization. In addition to providing accurate data for previously studied complexes, our CCSD(T)/CBS calculations fill in gaps arising from missing experimental thermochemical data for G interactions with all group I metals, and Rb^+^ or Cs^+^ bound to A, T, or U. Furthermore, accurate metal binding strengths for all group I metals to dimethylphosphate are provided herein for the first time, which affords a more realistic model than that used in experimental studies [neutral trimethylphosphate (Ruan et al., 2008)]. Thus, our work offers the first complete and accurate data set of the thermochemistry related to group I metal binding to each nucleic acid component.

### Accuracy of DFT methods for the binding strengths of group I metal -nucleic acid complexes involving direct coordination

**FIGURE 3:**
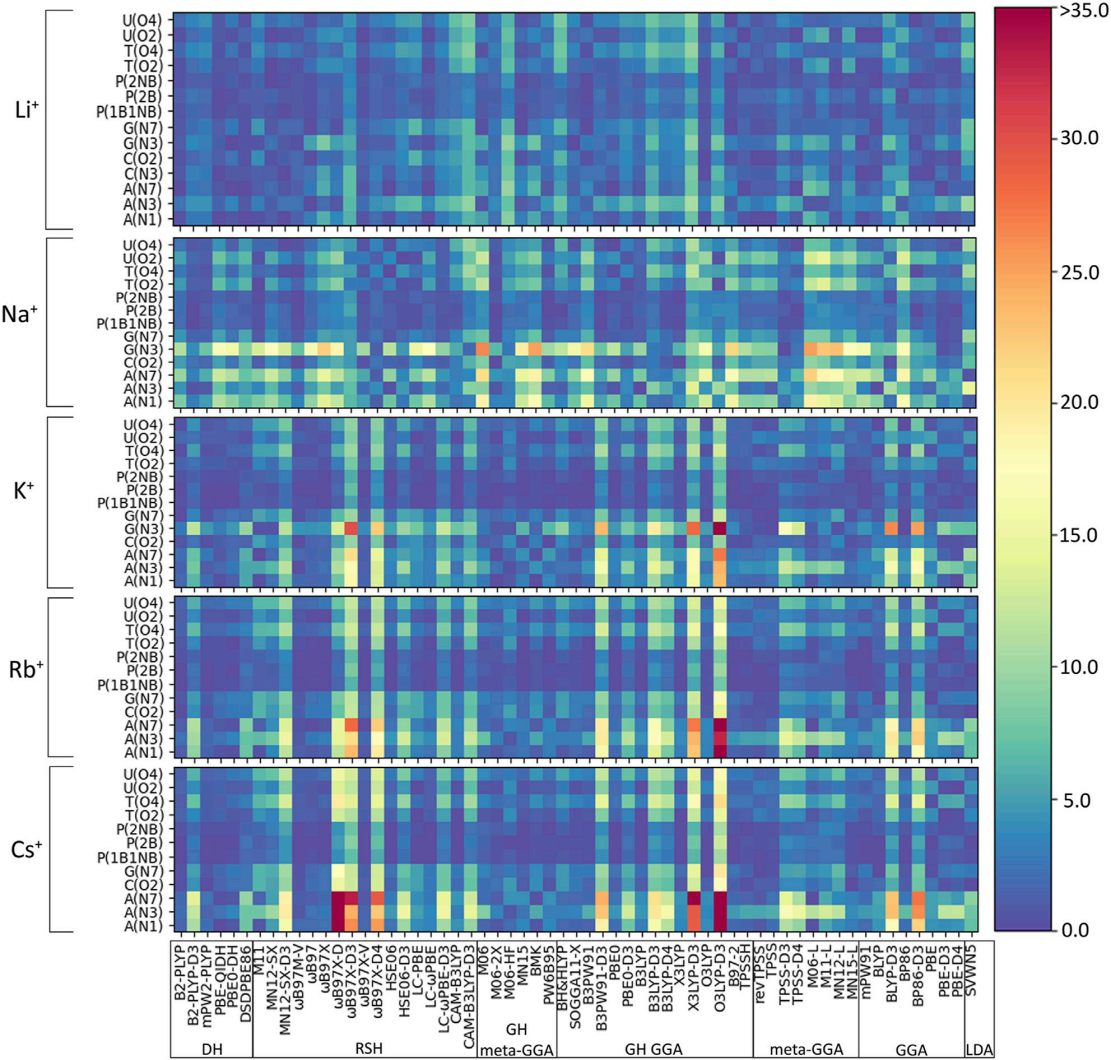
Percent errors (%) in DFT binding energies of group I metal–nucleic acid complexes calculated relative to CCSD(T)/CBS reference values, with small errors shown as dark blue shades and large errors highlighted by red shades.

As discussed in the Computational Methodology, the performance of 61 functionals (Table 1) in combination with def2-TZVPP is assessed against our newly generated CCSD(T)/CBS binding strengths for 64 directly coordinated group I metal–nucleic acid complexes (Figure 2). Initially, we consider the percent errors (PEs) in the DFT predicted binding strengths for each metal–nucleic acid component combination as a function of the functional (Figure 3). In general, PEs change both as a function of the complex and functional considered. Indeed, functional performance is dependent on the identity of the metal, nucleobase, and binding site. For example, interactions with the phosphate moiety tend to give rise to small errors regardless of metal (<5%), while weaker complexes, such as those involving N3 of G or A binding sites, give rise to larger deviations (>25%). Furthermore, larger errors occur as group 1 is descended, which underscores the importance of testing the reliability of DFT methods for different metals. Nevertheless, some functionals consistently exhibit the poorest performance across many complexes for all group I metals, such as O3LYP-D3(BJ) and X3LYP-D3(BJ).

**FIGURE 4:**
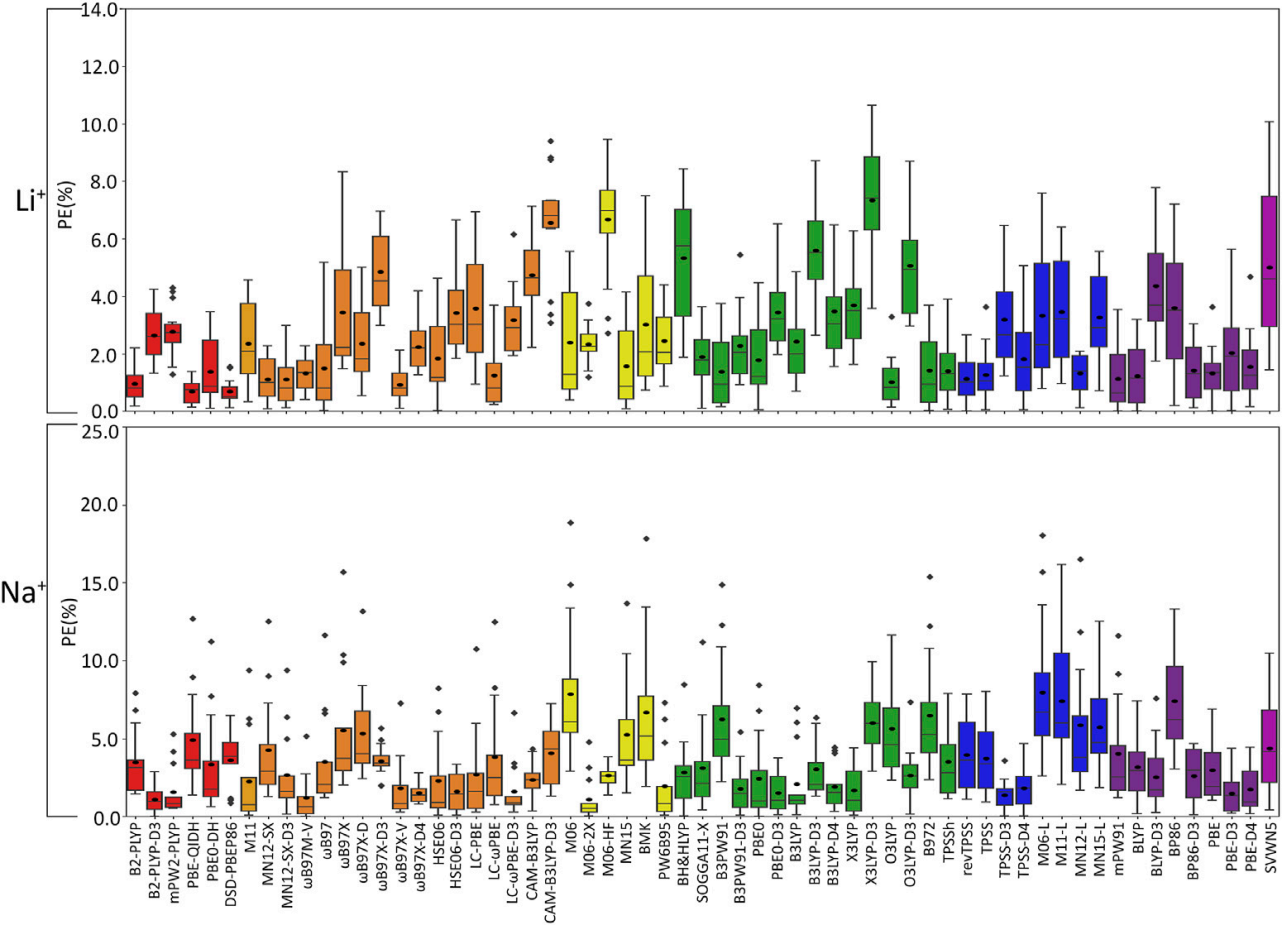
Boxplot plot statistics of the percent errors (%) in Li^+^ or Na^+^–nucleic acid DFT binding energies relative to CCSD(T)/CBS reference values, with the functionals sorted according to double-hybrids (red), RSH (orange), GH meta-GGA (yellow), GH GGA (green), meta-GGA (blue), GGA (purple), and LDA (magenta).

**FIGURE 5:**
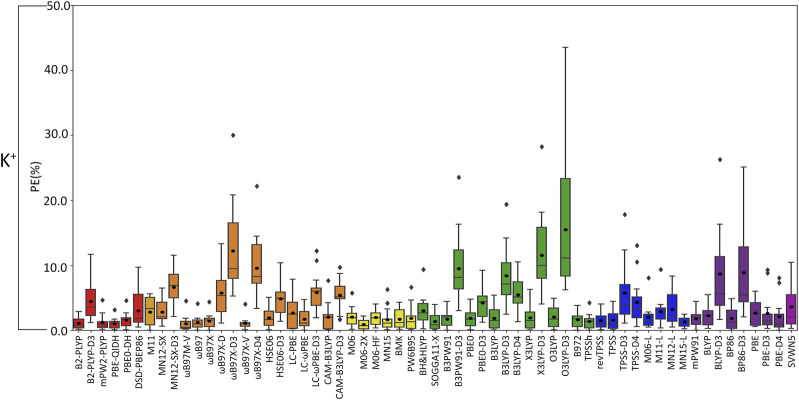
Boxplot plot statistics of the percent errors (%) in K^+^–nucleic acid DFT binding energies relative to CCSD(T)/CBS reference values, with the functionals sorted according to double-hybrids (red), RSH (orange), GH meta-GGA (yellow), GH GGA (green), meta-GGA (blue), GGA (purple), and LDA (magenta).

**FIGURE 6:**
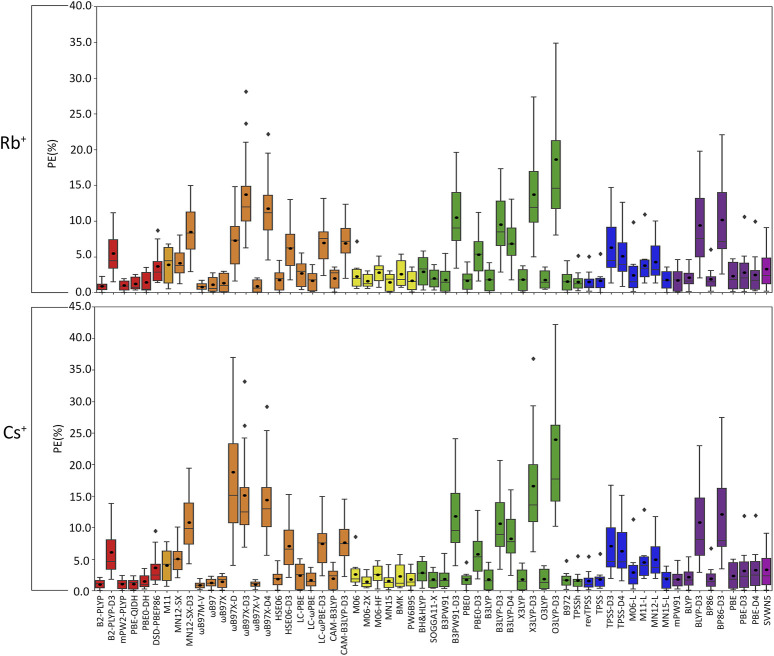
Boxplot plot statistics of the percent errors (%) in Rb^+^ or Cs^+^–nucleic acid DFT binding energies relative to CCSD(T)/CBS reference values, with the functionals sorted according to double-hybrids (red), RSH (orange), GH meta-GGA (yellow), GH GGA (green), meta-GGA (blue), GGA (purple), and LDA (magenta).

With future applications in mind and our goal to identify a robust functional regardless of group I metal–nucleic acid system considered, we gauge the reliability of functionals using both the PEs and unsigned errors (UEs) evaluated with respect to the CCSD(T)/CBS data across metal–nucleic acid complexes for each DFT method. Given the significant difference in the magnitude of the binding strength for various complexes, evaluation of both PEs and UEs ensures all interactions are more equally weighted. Functional recommendations were then selected based on boxplot statistics for both metrics. Despite the consideration of several metrics, we only report the mean percent error (MPE) and mean unsigned error (MUE) evaluated over complexes for each group I metal or over the entire data set in the main text for simplicity. We organize our discussion in the following subsections based on functional performance for the smallest (Li^+^) to largest (Cs^+^) metal, and subsequently consider functional performance over the entire group I, as well as the impact of neglecting counterpoise corrections (Figures 4–6, Supplementary Figures S2–S6; Supplementary Tables S3–S9).

(I) **Li** ^**+**^ **:** The most challenging nucleic acid complex to describe involving direct Li^+^ coordination for many functionals is A(N3) (Figure 3), which results in the most outliers in the box plot statistics (Supplementary Table S3). Regardless, when the statistics are considered over all complexes (Figure 4 and Supplementary Figure S2; Table 2), PBE-QIDH, DSD-PBEP86, and B2-PLYP demonstrate the best performance within the double-hybrid family for Li^+^–nucleic acid complexes (<1% MPE; <1 kcal/mol MUE). PBE-QIDH is marginally superior to the other double-hybrid functionals (0.7% ± 0.4% MPE; 0.4 ± 0.2 kcal/mol MUE), with a low spread in the data. The best RSH functional is ωB97X-V (0.9% ± 0.6% MPE; 0.6 ± 0.4 kcal/mol MUE), closely followed by ωB97M-V, MN12-SX-D3(BJ), and MN12-SX (∼1% MPEs; 0.7–0.9 kcal/mol MUEs). Within the GH meta-GGA family, the smallest average errors occur for MN15 (1.6% ± 1.4% MPE; 0.8 ± 0.6 kcal/mol MUE), followed by M06–2X and PW6B95, which offer similar performance to each other (∼2.4% MPE; ∼1.6 kcal/mol MUE), but larger maximum UEs than MN15. Among the 15 GH-GGA functionals explored in this study, O3LYP (1.0% ± 0.9% MPE; 0.6 ± 0.4 kcal/mol MUE) emerges as the top performer for Li^+^ complexes, with the smallest MPE, MUE, and standard deviations, as well as spread in the data, while SOGGA11-X, B3PW91, B97-2, and TPSSh show similar, slightly reduced accuracies (1.4%–1.9% MPE; 0.7–1.2 kcal/mol MUE). revTPSS is the best performing meta-GGA (1.1% ± 0.8%; 0.8 ± 0.6 kcal/mol), while TPSS and MN12-L are close runner-ups (1.3% MPE; 0.9 kcal/mol MUE). mPW91 is the only functional of the GGA family that stands out as a solid performer (1.1% ± 1.1% MPE; 0.7 ± 0.6 kcal/mol MUE; maximum errors = 3.5%; 1.6 kcal/mol). Multiple functionals that account for dispersion effects are reliable for Li^+^–nucleic acid complexes, including those that incorporate dispersion through corrections [e.g., ωB97M-V, ωB97X-V, and MN12-SX-D3(BJ)] or parameterization (e.g., M06–2X and MN15). When the best performers across different functional families are compared, the most reliable and robust methods recommended for investigating Li^+^–nucleic acid interactions are PBE-QIDH and ωB97X-V (X*, Table 2). Although PBE-QIDH was previously recommended as the overall top performing functional for Li^+^ systems based on only the most stable complex for each nucleic acid component (Boychuk et al., 2021), other functionals that were previously deemed accurate (ωB97, ωB97X-D, BP86-D3(BJ), PBE) are now replaced with those containing newer dispersion corrections (ωB97X-V), highlighting the importance of considering larger data sets that contain diverse complexes when identifying the top performers for thermochemical data of metal–nucleic acid complexes. For more cost-effective options, O3LYP leads to the lowest MPE and MUE, and the smallest maximum deviation (1.5 kcal/mol) compared to other functionals in the GH GGA, meta-GGA, and GGA families.

**TABLE 2:** Functionals identified as the top performer(s) for each functional family (X) and the best overall (recommended) functionals (X*, bold) for directly coordinated group I metal–nucleic acid complexes.^a^

Family	Functional	Li^+^	Na^+^	K^+^	Rb^+^	Cs^+^	Group 1
Double-Hybrid	B2-PLYP	X		X*	X	X*	
	B2-PLYP-D3		X*				
	mPW2-PLYP		X		X*	X	X*
	PBE-QIDH	X*		X	X	X	
	PBE0-DH						
	DSD-PBEP86	X					
RSH	M11						
	MN12-SX	X					
	MN12-SX-D3	X					
RSH meta-GGA	ωB97M-V	X	X	X	X*	X*	X*
RSH GGA	ωB97			X	X	X	
	ωB97X			X	X	X	
	ωB97X-D						
	ωB97X-D3						
	ωB97X-V	X*		X*	X	X*	X
	ωB97X-D4		X				
	HSE06			X			
	HSE06-D3		X*				
	LC-PBE						
	LC-ωPBE						
	LC-ωPBE-D3						
	CAM-B3LYP						
	CAM-B3LYP-D3						
GH meta-GGA	M06						
	M06–2X	X	X	X*	X	X	X
	M06-HF		X				
	MN15	X		X	X	X	
	BMK			X			
	PW6B95	X	X	X	X	X	X
GH GGA	BH&HLYP						
	SOGGA11-X	X		X		X	
	B3PW91	X					
	B3PW91-D3		X				
	PBE0				X		X
	PBE0-D3		X*				
	B3LYP				X	X	X
	B3LYP-D3						
	B3LYP-D4		X				
	X3LYP		X		X	X	
	X3LYP-D3						
	O3LYP	X*			X	X	
	O3LYP-D3						
	B97-2	X		X	X		
	TPSSh	X					
Local meta-GGA	revTPSS	X		X	X	X	X*
	TPSS	X		X	X	X	X*
	TPSS-D3		X*				
	TPSS-D4		X				
	M06-L						
	M11-L						
	MN12-L	X					
	MN15-L			X*	X*	X*	
Local GGA	mPW91	X		X	X	X	
	BLYP				X	X	X
	BLYP-D3						
	BP86			X			
	BP86-D3						
	PBE				X		X
	PBE-D3		X*				
	PBE-D4		X				
Local LDA	SVWN5						

^a^ Functional(s) indicated by an X were deemed to be reliable methods for a particular family for a given metal or over all group I metals. The overall top performing functionals that were found to be the most accurate technique(s) regardless of functional family for a given metal or over all group I metals are indicated by X*.

(II) **Na** ^**+**^ **:** In general, larger MPEs arise across functionals for Na^+^ relative to Li^+^–nucleic acid complexes (Figure 4), while the MUEs are more comparable between these lighter group I metals (Supplementary Figure S2). For Na^+^, the most challenging metal–nucleic acid complexes for the tested functionals to describe include A(N1–N6), A(N6–N7), and G(N3), which result in the most outliers according to the boxplot statistics (Figure 3; Supplementary Table S4). Among the double-hybrid functionals, B2-PLYP-D3(BJ) is the top performing method for predicting the binding energy of Na^+^–nucleic acid complexes (1.1% ± 0.9% MPE; 0.4 ± 0.3 kcal/mol MUE), followed by mPW2-PLYP (1.6% ± 1.6% MPE; 0.7 ± 0.5 kcal/mol MUE). Among the 17 functionals within the RSH family, HSE06-D3(BJ) is the most reliable (1.6% ± 1.3% MPE; 0.7 ± 0.3 kcal/mol MUE), with no outliers for either metric in the boxplot statistics. ωB97M-V and ωB97X-D4 are close runners-ups (1.2%–1.5% MPE; 0.4–0.9 kcal/mol), although result in larger maximum errors (≤5.2%; ≤1.9 kcal/mol). M06-2X, which was deemed reliable for Li^+^, stands out as the best GH meta-GGA functional for Na^+^ interactions, with the smallest magnitude and spread in the deviations (1.1% ± 1.4% MPE; 0.4 ± 0.4 kcal/mol MUE), albeit with several outliers for PE. M06-2X is followed by M06-HF and PW6B95, which have similar errors to each other (2.0%–2.6% MPE; 0.7–1.4 kcal/mol MUE), but larger maximum UEs than M06-2X (2.2–3.7 kcal/mol). Unlike Li^+^, PBE0-D3(BJ) and X3LYP from the GH GGA family emerge as the top performing functionals (1.5%–1.7% MPE; 0.6 kcal/mol MUE), followed by B3PW91-D3(BJ) and B3LYP-D4, which have slightly reduced accuracies (1.8%–1.9% MPE; 0.7–0.8 kcal/mol MUE) and reliability (SDs of 1.5%–1.7% MPE; 0.4–0.5 kcal/mol MUE). TPSS-D3(BJ) is the meta-GGA that leads to the least errors (1.4% ± 1.0% MPE; 0.6 ± 0.3 kcal/mol MUE), while TPSS-D4 is a close runner-up (1.8% ± 1.4% MPE; 0.8 ± 0.5 kcal/mol MUE). Of the GGA functionals, only the D3(BJ) and D4-corrected versions of PBE offer solid performance (1.5%–1.7% MPE; 0.6–0.8 kcal/mol MUE), with small standard deviations (1.3%–1.4% MPE; 0.4–0.5 kcal/mol). Like Li^+^, many of the reliable functionals for Na^+^ complexes account for dispersion through corrections (ωB97M-V and ωB97X-V) or parameterization (M06-2X), with both ωB97M-V and M06-2X being highly dependable for both metals and ωB97X-V offering reasonable reliability.

 Overall, the recommended functionals that consistently offer the highest accuracy for Na^+^–nucleic acid complexes are B2PLYP-D3(BJ) and HSE06-D3(BJ) (X*, Table 2). While neither functional is the best option for Li^+^, B2PLYP-D3(BJ) still offers reasonable performance for the smallest group I metal, with only moderately larger errors (2.7% MPE; 1.8 kcal/mol MUE) than for Na^+^. In contrast, although the top-performing HSE06-D3(BJ) for Na^+^ fails to accurately describe Li^+^ (3.4% MPE; 2.2 kcal/mol MUE), ωB97M-V works well for both metals (1.2%–1.3% MPE; 0.4–0.9 kcal/mol MUE). Despite more inexpensive options not being consistent among the lighter group I metals, the PBE0-D3(BJ) GH GGA functional, TPSS-D3(BJ) from the meta-GGA family, and PBE-D3(BJ) from the GGA family are all reliable for Na^+^–nucleic acid complexes, with low MPEs and MUEs, and small maximum deviations (<5%; ≤1.4 kcal/mol).

(III) **K** ^**+**^ **:** In general, the magnitude of error from CCSD(T)/CBS data increases for K^+^ compared to the lighter metals (Figure 3), with over double the maximum PE (∼45% PE K^+^; <19% PE Li^+^, Na^+^; Figures 4, 5; Supplementary Tables S3–S5), which is largely due to G and A complexes formed at the N3 position. These results may be attributed to the weaker binding energies between K^+^ and each nucleic acid component (by ∼25–49 kcal/mol compared to Li^+^ and ∼12–24 kcal/mol with respect to Na^+^; Supplementary Table S2). In fact, three functionals [i.e., O3LYP-D3(BJ), X3LYP-D3(BJ), and ωB97X-D3(BJ)] have MPEs greater than 10% for K^+^. Among the double-hybrids, B2-PLYP and PBE-QIDH demonstrate similar accuracy (1.1%–1.2% MPE; 0.3 kcal/mol MUE), with low maximum PEs (∼3%). B2-PLYP provides marginally better descriptions of K^+^–nucleic acid interactions, which is also a reliable double-hybrid functional for Li^+^ and the dispersion-corrected variant was one of the best functionals for Na^+^ complexes. Within the RSH family, four of the six functionals in the ωB97 series (ωB97, ωB97X, ωB97M-V, and ωB97X-V) offer excellent performance (1.1%–1.4% MPE; 0.3 kcal/mol MUE) that is comparable to the double-hybrid functionals, with ωB97X-V demonstrating slightly enhanced accuracy (1.1% ± 1.0% MPE; 0.3 ± 0.1 kcal/mol MUE). HSE06 also performs well, with no outliers in the boxplot statistics, but a larger spread in the data compared to ωB97X-V (1.9% ± 1.5% MPE; 0.4 ± 0.2 kcal/mol MUE). Among the ωB97 series of functionals, ωB97M-V and ωB97X-V both demonstrate reasonable reliability across Li^+^, Na^+^, and K^+^ complexes. The best performing functional of the GH meta-GGA family for Na^+^ complexes is M06-2X (0.9% ± 0.7% MPE; 0.3 ± 0.3 kcal/mol MUE). MN15, PW6B95, and BMK are the next best functionals in this family (1.7%–1.8% MPE; 0.4–0.5 kcal/mol MUE), which are less reliable than M06–2X due to outliers and larger spreads in the data according to the boxplot statistics. SOGGA11-X and B97-2 of the GH GGA family consistently demonstrate accurate performance across all K^+^ complexes (1.4%–1.6% MPE; 0.4 kcal/mol MUE). Both SOGGA11-X and B97-2 show reasonable accuracy for Li^+^ (∼1–2% MPE; ∼1 kcal/mol MUE), but not Na^+^ (∼3–7% MPE; ∼1–3 kcal/mol MUE). Within the meta-GGA family, revTPSS, MN15-L, and TPSS are the most reliable methods (1.3%–1.6% MPE; 0.4 kcal/mol MUE), with MN15-L having slightly better performance mainly due to a smaller spread in the data (1.3% ± 0.8% MPE; 0.4 ± 0.3 kcal/mol MUE). In terms of the GGA functionals, although mPW91 and BP86 have minimal average (1.8% MPE; 0.4–0.6 kcal/mol MUE) and maximum (<5.0%) errors, mPW91 emerges as the most reliable option due to the smallest spread in the data over both metrics. Some functionals identified for K^+^ within the meta-GGA (revTPSS) and GGA (mPW91) families are also accurate for Li^+^, but less reliable for Na^+^ (4.0% MPE; 1.8 kcal/mol MUE).

 Based on low MPEs, MUEs, and minor maximum errors, the recommended functionals for investigating K^+^–nucleic acid interactions are B2-PLYP, ωB97X-V, and M06-2X (X*, Table 2). ωB97X-V and M06-2X, which each account for dispersion, also offer reasonable reliability for Li^+^ and Na^+^. While B2-PLYP is also an accurate double-hybrid functional for Li^+^, the corresponding dispersion-corrected variant was one of the recommended methods for Na^+^ complexes. MN15-L is also available as a computationally affordable option for K^+^–nucleic acid complexes.

(IV) **Rb** ^**+**^ **:** As seen with K^+^, certain functionals emerge as having substantially larger errors for Rb^+^–nucleic acid interactions (maximum PE of ∼22–42%), including O3LYP-D3(BJ), X3LYP-D3(BJ), ωB97X-D3(BJ), and ωB97X-D4, which is mainly attributed to the A complexes (Figures 3, 6; Supplementary Table S6). Indeed, the number of functionals exceeding an MPE of 10% doubles for Rb^+^ (6; Figure 6) compared to K^+^ (3; Figure 5). mPW2-PLYP and B2-PLYP are the top performers within the double-hybrid family (∼1% MPE; 0.2 kcal/mol MUE; Figure 6 and Supplementary Figure S3), closely followed by PBE-QIDH (1.2% ± 0.9% MPE; 0.4 ± 0.2 kcal/mol MUE). mPW2-PLYP emerges as the best double-hybrid functional (maximum errors = 1.9%; 0.4 kcal/mol). The most accurate functional from the RSH family is ωB97M-V (0.8% ± 05% MPE; 0.2 ± 0.1 kcal/mol MUE), followed by ωB97X-V, ωB97, and ωB97X (≤1.3% MPE; 0.2–0.3 kcal/mol MUE). Although multiple functionals from the ωB97 series are recommended for Li^+^, Na^+^, K^+^, and Rb^+^, others from this category (ωB97X-D3(BJ) and ωB97X-D4) exhibit large errors for Rb^+^ and K^+^. MN15 is the most reliable functional of the GH meta-GGA family (1.5% ± 1.2% MPE; 0.4 ± 0.2 kcal/mol MUE), closely followed by M06-2X (1.6% ± 0.9% MPE; 0.5 ± 0.3 kcal/mol MUE) and PW6B95 (1.7% ± 1.3% MPE; 0.4 ± 0.2 kcal/mol MUE). Within the GH GGA family, O3LYP is the most accurate (1.8% ± 1.2% MPE; 0.5 ± 0.2 kcal/mol; maximum errors = 3.6%; 0.7 kcal/mol), while B3LYP, X3LYP, PBE0, and B97-2 offer comparable performance to each other as viable alternatives (1.6%–1.8% MPE; 0.4 kcal/mol MUE). From the meta-GGA family, MN15-L is more reliable (1.8% ± 1.3% MPE; 0.4 ± 0.2 kcal/mol MUE) than TPSS and revTPSS (1.5%–1.6% MPE; 0.4 kcal/mol) due to the absence of outliers and lower maximum errors, with MN15-L also being one of the recommended methods for K^+^. Like Li^+^ and K^+^, mPW91 emerges as the top performer of the GGA family (1.7% ± 1.5% MPE; 0.4 ± 0.3 kcal/mol MUE), with no outliers for either metric. For Rb^+^ complexes, mPW91 is followed by BLYP and PBE (2.1%–2.3% MPE; 0.6–0.7 kcal/mol MUE). Multiple accurate functionals for Rb^+^ complexes account for dispersion through corrections (ωB97M-V and ωB97X-V) or parameterization (M06-2X).

 Overall, despite being more challenging to describe than the metals discussed thus far, there are many reliable functionals for exploring Rb^+^–nucleic acid interactions. Our recommendations include mPW2-PLYP and ωB97M-V (X*, Table 2), which display low MPEs, MUEs, and maximum deviations (≤1.9%; 0.4 kcal/mol). Although not the overall best double-hybrid functional for each metal discussed previously, mPW2-PLYP performs very well for K^+^ complexes and offers reasonable agreement to CCSD(T)/CBS data for Li^+^ and Na^+^. Additionally, ωB97M-V has demonstrated consistently small errors for Li^+^, Na^+^, K^+^, and Rb^+^. As a cost-effective alternative for Rb^+^–nucleic acid complexes, MN15-L offers low MPEs, MUEs, and reasonable maximum deviations (3.6%; 0.7 kcal/mol).

(V) **Cs** ^**+**^ **:** Cs^+^ demonstrates the largest errors among group I metals (maximum PE of 59.0%; Figure 6; Supplementary Table S7). Indeed, 10 functionals result in significantly large MPEs (>10%). This highlights the increased sensitivity of DFT with increased size of the metal and decreased magnitude of the binding strength. Similar to K^+^ and Rb^+^, the A(N3) complex results in the most deviations from CCSD(T)/CBS data (Figure 3; Supplementary Table S7). Within the double-hybrid family, B2-PLYP is the best performer (1.0% ± 0.7% MPE; 0.2 ± 0.1 kcal/mol MUE; maximum errors = 2.1%; 0.5 kcal/mol; Figure 6). Although B2-PLYP was also recommended for K^+^ and offers small errors for Li^+^ and Rb^+^, the performance is not as good as other double-hybrids for Na^+^, requiring the addition of a dispersion correction. The next best performers in the double-hybrid family are mPW2-PLYP (1.1% MPE; 0.2 kcal/mol MUE), which is reasonable for all group I metals, and PBE-QIDH (1.1% MPE; 03 kcal/mol MUE), which is recommended for Li^+^, reliable for K^+^ and Rb^+^, but a poor performer for Na^+^. The most reliable functionals for Cs^+^ among the RSH family are ωB97M-V and ωB97X-V (0.9%–1.0% MPE; 0.2 kcal/mol), while ωB97 and ωB97X are the next best performers (1.2%–1.4% MPE; 0.3 kcal/mol). ωB97M-V was one of the recommended functionals for Rb^+^, while ωB97X-V was recommended for Li^+^ and K^+^. Nevertheless, the performance of both ωB97M-V and ωB97X-V is excellent for all metals, even though HSE06-D3(BJ) was the best RSH family member for Na^+^. Amongst the GH meta-GGA functionals, MN15 demonstrates the smallest deviations from CCSD(T)/CBS (1.5% MPE; 0.3 kcal/mol MUE), followed by M06-2X and PW6B95 (1.5%–1.8% MPE; 0.4 kcal/mol). Indeed, MN15 demonstrates a slightly smaller spread in the data and the lowest maximum UE (0.6 kcal/mol). Like Li^+^ (the lightest group I metal), O3LYP offers consistent and reliable performance for Cs^+^ (the heaviest group I metal) within the GH GGA family (1.9% ± 1.5% MPE; 0.4 ± 0.2 kcal/mol MUE), closely followed by B3LYP, X3LYP, and SOGGA11-X (1.8% MPE; 0.4–0.5 kcal/mol MUE). Within the meta-GGA family, three functionals (revTPSS, TPSS, and MN15-L) offer the best performance (1.6%–1.8% MPE; 0.4 kcal/mol MUE), with MN15-L emerging as the top performer due to the smallest standard deviations (1.4% MPE; 0.1 kcal/mol) and maximum errors (3.9%; 0.4 kcal/mol). Within the GGA family, mPW91 is the most accurate functional (1.8% ± 1.3% MPE; 0.4 ± 0.2 kcal/mol MUE), followed by BLYP (2.2% ± 1.9% MPE; 0.5 ± 0.3 kcal/mol MUE). Both methods are also among the most reliable GGA functionals for Rb^+^.

**FIGURE 7:**
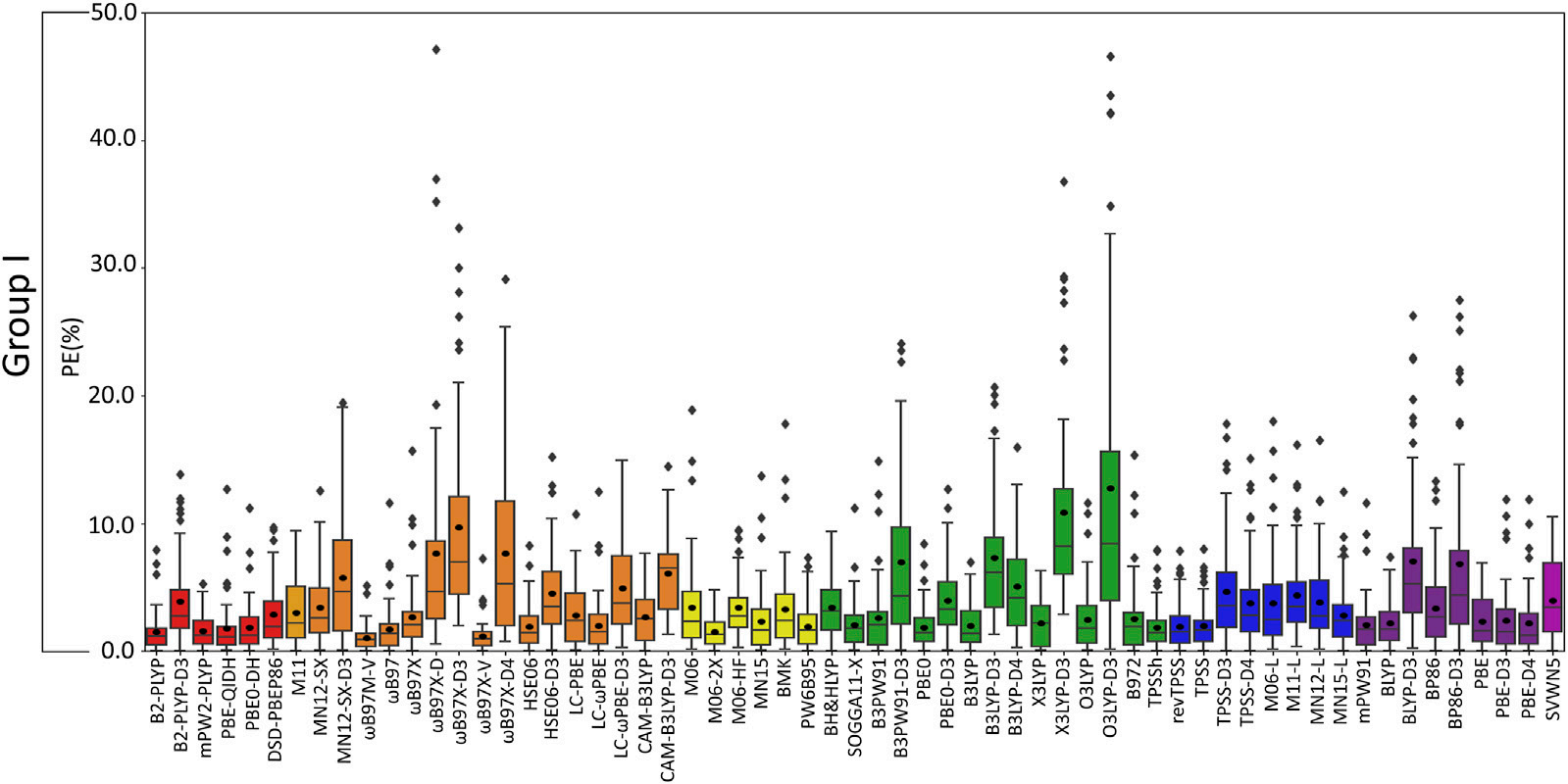
Boxplot plot statistics of the percent errors (%) for group I metal–nucleic acid DFT binding energies relative to CCSD(T)/CBS reference values, with the functionals sorted according to double-hybrids (red), RSH (orange), GH meta-GGA (yellow), GH GGA (green), meta-GGA (blue), GGA (purple), and LDA (magenta).

 Overall, the recommended functionals for investigating Cs^+^–nucleic acid interactions are B2-PLYP, ωB97M-V, and ωB97X-V (X*, Table 2), which demonstrate consistent and accurate performance across all complexes, including low maximum errors (≤2.1%; ≤0.5 kcal/mol). As mentioned above, B2-PLYP yields small deviations from CCSD(T)/CBS data for all metals except Na^+^, although the MUE across Na^+^ complexes is still below 2 kcal/mol. Both ωB97M-V and ωB97X-V, which include the more recently designed VV10 dispersion correction (Mardirossian and Head-Gordon, 2017), have emerged as solid performers across all group I metal–nucleic acid complexes. In terms of cost-effective options for Cs^+^ complexes, MN15-L is deemed reliable.

(VI) **Although the Top-Performing Functionals Can Vary with Metal, Some Methods Prevail as Being Reliable Across Group I:** Thus far, variation in functional performance has been seen for each group I metal as well as between the lighter (Li^+^ and Na^+^) and heavier (K^+^, Rb^+^, and Cs^+^) metals (Figures 3–6). Nevertheless, a robust method is required that offers a reasonable description of all group I metal–nucleic acid complexes. This is important for understanding, for example, problems related to metal competition in biology, diseases, energy storage materials, and nucleic acid sensors. Therefore, in this section, functional performance is compared over all Li^+^, Na^+^, K^+^, Rb^+^, and Cs^+^–nucleic acid complexes that involve direct metal coordination (Figures 2, 7 and Supplementary Figure S4). Among the six double-hybrid functionals, mPW2-PLYP is the most accurate across all group I metal–nucleic acid binding strengths (1.6% ± 1.3% MPE; 0.7 ± 0.7 kcal/mol MUE; maximum errors = 5.3%; 2.7 kcal/mol). While mPW2-PLYP was not the best performing double-hybrid for the lighter metals, this method still offers a reasonable description across complexes involving Li^+^ or Na^+^ (≤2.8% MPE; ≤1.8 kcal/mol MUE). ωB97M-V emerges as the best performer over group I within the RSH family (1.1% ± 1.0% MPE; 0.4 ± 0.4 kcal/mol MUE), as well as Rb^+^ and Cs^+^, closely followed by ωB97X-V (1.1% ± 1.2% MPE; 0.4 ± 0.4 kcal/mol MUE), which is one of the best functionals for Li^+^ and K^+^. Although slightly less accurate for Na^+^ complexes, both ωB97M-V and ωB97X-V offer reasonable binding energies (<1 kcal/mol MUE deviation from CCSD(T)/CBS). Within the GH meta-GGA family, M06-2X demonstrates the smallest errors (1.5% ± 1.1% MPE; 0.7 ± 0.6 kcal/mol MUE), with PW6B95 being a close runner-up (2.0% ± 1.7% MPE; 0.7 ± 0.7 kcal/mol MUE), albeit having more outliers. M06-2X was one of the best functionals for K^+^ and offers a reasonable description for Li^+^, Na^+^, Rb^+^, and Cs^+^ (≤2.3% MPE; ≤1.5 kcal/mol MUE). The most reliable functionals of the GH GGA family are B3LYP and PBE0 (≤2.0% MPE; ≤0.8 kcal/mol MUE). These functionals are generally more reliable for the heavier metals (K^+^, Rb^+^, and Cs^+^), but still afford reasonable accuracy for the lighter metals (Li^+^ and Na^+^; ∼2% MPE; ∼1–2 kcal/mol MUE). Both revTPSS and TPSS offer the best performance within the meta-GGA family (1.9%–2.0% MPE; 0.8 kcal/mol), while BLYP and PBE emerge as the best GGA functionals (2.2%–2.3% MPE; 0.9 kcal/mol; maximum errors ≤7.4%; ≤3.3 kcal/mol).

 Overall, the recommended functionals for exploring group I metal–nucleic acid interactions are mPW2-PLYP and ωB97M-V (X*, Table 2), which demonstrate the smallest MPEs and MUEs, and low maximum errors (≤5.3%; ≤2.7 kcal/mol). Indeed, ωB97M-V has been identified as the most promising functional for main-group chemistry (Mardirossian and Head-Gordon, 2017), and is deemed one of the most accurate functionals for exploring transition metal chemistry (Chan et al., 2019). Since both recommended functionals are from more expensive families, cost-effective methods must also be identified to explore group I metal interactions using larger models that are required to accurately study biosystems. Although the most popular DFT method (B3LYP) offers reasonable computational performance on average for group I metals, B3LYP leads to larger maximum errors (7.0%; 2.7 kcal/mol) compared to the top-performing functionals, as well as a higher spread in the boxplot data. Indeed, cost-effective revTPSS and TPSS are on average the most reliable across group I (X*, Table 2), with MPEs and MUEs comparable to the best performing functionals, albeit having larger spreads and more outliers in the boxplot data than the more computationally intense methods.

(VII) **Impact of Counterpoise Corrections on Group I Metal–Nucleic Acid Binding Energies:** Although all results discussed thus far include counterpoise corrections, such corrections are not always computationally feasible, especially when using large models that are necessary to investigate complex biosystems or biomaterials. To ensure that functional performance remains unchanged regardless of whether counterpoise corrections are included, the counterpoise uncorrected and corrected binding energies are compared for the most reliable functionals for each group I metal and over all group I metals (Supplementary Figures S5, S6; Supplementary Table S9). In general, there is a slight increase in deviations from the CCSD(T)/CBS binding energies when the counterpoise corrections are not included in the DFT binding strengths. Nevertheless, the differences between the counterpoise-corrected and uncorrected MPEs and MUEs are ≤1.5% and ≤0.6 kcal/mol, respectively, which correlates with previous literature reporting a minimal BSSE impact for similar systems (Boychuk et al., 2021). This suggests that counterpoise corrections can be neglected when implementing larger computational models while minimally impacting the accuracy of the resulting DFT description of group I metal–nucleic acid interactions.

## Conclusion

In summary, a new database of accurate gas-phase CCSD(T)/CBS group I metal–nucleic acid binding energies has been generated, which has otherwise proven to be challenging to compile experimentally due to measurement sensitivities and nucleobase tautomerizations. Moreover, this study provides highly accurate interaction energies between group I metals and G as well as between heavier metals (Rb^+^ and Cs^+^) and A, T, or U for which no experimental thermochemical data is currently available. Subsequently, the performance of 61 DFT functionals was tested across 64 directly coordinated group I metal–nucleic acid complexes. Although functional performance can vary with the metal, nucleic acid component, and binding site, reliable methods across all group I metal–nucleic acid interactions came to light. Indeed, mPW2-PLYP and ωB97M-V demonstrate remarkably small MPEs and MUEs (≤1.6%; <1.0 kcal/mol) and low spreads in the data based on boxplot statistics. If more computationally efficient methods are required, revTPSS and TPSS offer reasonable reliability, albeit having larger spreads and more outliers in the boxplot statistics compared to the more computationally intensive functionals. Counterpoise corrections are found to have negligible effects on the accuracy of the top-performing functionals for group I metal–nucleic acid binding energies (≤1.5% and ≤0.6 kcal/mol), which is promising for investigations that require the implementation of larger models that render counterpoise corrections unfeasible. Overall, the accurate methods identified in our work can be used in the future to understand the role of metals in ecological and human biology, to generate new therapeutics, to explore the design of novel chemical technologies (e.g., new materials for energy storage and nucleic acid sensors that target metal contaminants in the environment or human body), and to aid the design of new computational methods that permit broader investigations of group I metal–nucleic acid interactions.

## Data Availability

The original contributions presented in the study are included in the article/Supplementary Material. Further inquiries can be directed to the corresponding author.
