# Supplementary material for: Generation of an accurate CCSD(T)/CBS data set and assessment of DFT methods for the binding strengths of group I metal–nucleic acid complexes
Source: Front Chem. 2023 Nov 20;11:1296787. doi: 10.3389/fchem.2023.1296787 (PMC10694745; doi:10.3389/fchem.2023.1296787)
Supplement: Supplementary file 1 [file DataSheet1.pdf]

Supplementary Material

Generation of an Accurate CCSD(T)/CBS Data Set and Assessment of DFT Methods for the Binding Strengths of Group I Metal–Nucleic Acid Complexes

Briana T. A. Boychuk, Sarah P. Meyer, and Stacey D. Wetmore*
***Correspondance:** Stacey D. Wetmore: stacey.wetmore@uleth.ca

Table of Contents
(23 pages)

Table S1. Metal–nucleic acid coordination distances (Å) between each group I metal (Li^+, Na^+, K^+, Rb^+, or Cs^+) and nucleic acid component (A, C, T, G, U, or P)……………………………S3

Table S2. CCSD(T)/CBS binding strengths of group I metal–nucleic acid complexes in kcal/mol……………………………………………………………………………….…………S4

Table S3. MPEs (%) and MUEs (kcal/mol) in DFT binding strengths along with corresponding standard (SD), minimum (Min), and maximum (Max) deviations evaluated across Li^+–nucleic acid complexes for each functional relative to CCSD(T)/CBS reference values, as well as outliers based on the boxplot analysis………………………..……………………………………..S5

Table S4. MPEs (%) and MUEs (kcal/mol) in DFT binding strengths along with corresponding standard (SD), minimum (Min), and maximum (Max) deviations evaluated across Na^+–nucleic acid complexes for each functional relative to CCSD(T)/CBS reference values, as well as outliers based on the boxplot analysis……………………………………………………..…………..S7

Table S5. MPEs (%) and MUEs (kcal/mol) in DFT binding strengths along with corresponding standard (SD), minimum (Min), and maximum (Max) deviations evaluated across K^+–nucleic acid complexes for each functional relative to CCSD(T)/CBS reference values, as well as outliers based on the boxplot analysis..……..……………………………………………….……....S9

Table S6. MPEs (%) and MUEs (kcal/mol) in DFT binding strengths along with corresponding standard (SD), minimum (Min), and maximum (Max) deviations evaluated across Rb^+–nucleic acid complexes for each functional relative to CCSD(T)/CBS reference values, as well as outliers based on the boxplot analysis……………………..……………..……………………..S11

Table S7. MPEs (%) and MUEs (kcal/mol) in DFT binding strengths along with corresponding standard (SD), minimum (Min), and maximum (Max) deviations evaluated across Cs^+–nucleic acid complexes for each functional relative to CCSD(T)/CBS reference values, as well as outliers based on the boxplot analysis……………………………………………………………..S13

Table S8. MPEs (%) and MUEs (kcal/mol) in DFT binding strengths along with corresponding standard (SD), minimum (Min), and maximum (Max) deviations evaluated across group I metal–nucleic acid complexes for each functional relative to CCSD(T)/CBS reference values, as well as outliers based on the boxplot analysis………………………..……………………………...S15

Table S9. MPEs and MUEs for each DFT functional (with and without counterpoise corrections) relative to CCSD(T)/CBS reference values for group I metal complexes……….……………S18

Figure S1. CCSD(T)/CBS//MP2/def2-TZVPP characterized P(2B) complex between each metal and the dimethylphosphate model, which includes an additional contact with the nonbridging oxygen for all metals except Li^+………..……………………………………….………..S19

Figure S2. Boxplot plot statistics of the unsigned errors (kcal/mol) in Li^+ or Na^+–nucleic acid DFT binding energies relative to CCSD(T)/CBS reference values, with the functionals sorted according to double-hybrids (red), RSH (orange), GH meta-GGA (yellow), GH GGA (green), meta-GGA (blue), GGA (purple), and LDA (magenta)… ……………….……………………S20

Figure S3. Boxplot plot statistics of the unsigned errors (kcal/mol) in K^+, Rb^+ or Cs^+–nucleic acid DFT binding energies relative to CCSD(T)/CBS reference values, with the functionals sorted according to double-hybrids (red), RSH (orange), GH meta-GGA (yellow), GH GGA (green), meta-GGA (blue), GGA (purple), and LDA (magenta)… …..……………….………S21

Figure S4. Boxplot plot statistics of the unsigned errors (kcal/mol) in group I metal–nucleic acid DFT binding energies relative to CCSD(T)/CBS reference values, with the functionals sorted according to double-hybrids (red), RSH (orange), GH meta-GGA (yellow), GH GGA (green), meta-GGA (blue), GGA (purple), and LDA (magenta)……………...……………...…………S22

Figure S5: MPEs (%) in the uncorrected (solid) and counterpoise-corrected (striped) DFT binding energies relative to the CCSD(T)/CBS reference values for the top-performing functionals for each metal and over all group I…………………..…………………………S22

Figure S6: MUEs (kcal/mol) in the uncorrected (solid), and counterpoise-corrected (striped) DFT binding energies relative to the CCSD(T)/CBS reference values for the top-performing functionals for each metal and over all group I…...……………………………………………S23

Table S1. Metal–nucleic acid coordination distances (Å) between each group I metal (Li^+, Na^+, K^+, Rb^+, or Cs^+) and nucleic acid component (A, C, T, G, U, or P).[a]

Binding Site		Li^+	Na^+	K^+	Rb^+	Cs^+
A(N6–N7)	X···N6	2.081	2.467	2.884	3.032	3.195
	X···N7	1.961	2.349	2.777	2.947	3.138
A(N1–N6)	X···N1	1.965	2.360	2.765	2.923	3.074
	X···N6	2.042	2.460	2.896	3.057	3.236
A(N3)	X···N3	1.887	2.283	2.696	2.862	3.013
G(O6–N7)	X···O6	1.927	2.278	2.635	2.782	2.923
	X···N7	2.030	2.387	2.790	2.951	3.121
G(N2–N3)	X···N2	2.050	2.437	2.921		
	X···N3	1.949	2.352	2.783		
C(O2–N3)	X···O2	1.859	2.209	2.560	2.707	2.836
	X···N3	2.058	2.471	2.911	3.082	3.268
C(N3–N4)	X···N3	1.938				
	X···N4	2.114				
T(O4)	X···O4	1.706	2.100	2.490	2.651	2.798
T(O2)	X···O2	1.709	2.103	2.497	2.659	2.813
U(O4)	X···O4	1.710	2.103	2.495	2.656	2.808
U(O2)	X···O2	1.713	2.109	2.506	2.669	2.826
P(2NB)	X···O(NB)	1.860	2.212	2.545	2.684	2.790
	X···O(NB)	1.862	2.212	2.545	2.684	2.790
P(1B1NB)	X···O(B)	1.875	2.253	2.625	2.772	2.926
	X···O(NB)	1.784	2.121	2.446	2.582	2.669
P(2B)	X···O(B)	1.800	2.400	2.802	2.915	3.103
	X···O(B)	1.800	2.606	2.801	2.946	3.103
	X···O(NB)[b]		2.191	2.510	2.648	2.726

[a]Empty spaces within the table indicate that specific metal–nucleic acid complex could not be isolated. See Figure 2 for definitions of complexes considered in the present work. [b]Coordination to a non-bridging oxygen in the dimethylphosphate model also occurs for all metals except Li^+ as shown in Figure S1.

Table S2. CCSD(T)/CBS binding strengths of group I metal–nucleic acid complexes in kcal/mol.[a]

Binding Site	Li^+	Na^+	K^+	Rb^+	Cs^+
A(N6–N7)	–51.1	–36.0	–19.4	–16.5	–14.2
A(N1–N6)	–48.9	–34.7	–19.6	–17.1	–15.1
A(N3)	–44.4	–31.5	–18.0	–15.7	–13.8
G(O6–N7)	–73.0	–58.0	–39.9	–36.4	–33.4
G(N2–N3)	–40.6	–26.1	–11.3		
C(O2–N3)	–69.3	–53.8	–36.6	–33.2	–30.5
C(N3–N4)	–47.5				
T(O4)	–50.4	–36.3	–23.8	–21.4	–19.5
T(O2)	–48.8	–34.9	–22.7	–20.4	–18.6
U(O4)	–50.3	–36.4	–24.1	–21.7	–19.8
U(O2)	–46.7	–33.2	–21.3	–19.1	–17.3
P(2NB)	–163.9	–140.3	–115.3	–110.0	–105.5
P(1B1NB)	–157.8	–135.4	–111.8	–106.8	–102.7
P(2B)	–143.3	–133.3	–110.6	–105.8	–102.0

[a]Empty spaces within the table indicate that specific metal–nucleic acid complex could not be isolated. See Figure 2 for definitions of complexes considered in the present work.

Table S3. MPEs (%) and MUEs (kcal/mol) in DFT binding strengths along with corresponding standard (SD), minimum (Min), and maximum (Max) deviations evaluated across Li^+–nucleic acid complexes for each functional relative to CCSD(T)/CBS reference values, as well as outliers based on the boxplot analysis.

Family	Functional	MPE	SD	Min	Max	Outliers[a]	MUE	SD	Min	MAX	Outliers[a]
**Double-Hybrid**	B2-PLYP	1.0	0.6	0.2	2.2		0.7	0.5	0.1	1.9	
	B2-PLYP-D3	2.7	0.9	1.3	4.2		1.8	0.7	0.9	3.7	P(1B1NB,2B)
	mPW2-PLYP	2.8	0.9	1.3	4.3	P(2NB), T(O4), U(O4), A(N3)	1.8	0.5	1.2	2.7	
	PBE-QIDH	0.7	0.4	0.1	1.4		0.4	0.2	0.1	0.8	
	PBE0-DH	1.4	1.1	0.1	3.5		0.9	0.7	0.0	1.9	
	DSD-PBEP86	0.7	0.4	0.1	1.5	U(O2)	0.5	0.4	0.1	1.5	
**RSH**	M11	2.3	1.5	0.3	4.6		1.6	1.0	0.2	3.3	
	MN12-SX	1.1	0.8	0.1	2.3		0.7	0.5	0.0	2.1	P(2B)
	MN12-SX-D3	1.1	1.0	0.1	3.0		0.7	0.5	0.1	1.5	
**RSH meta-GGA**	ωB97M-V	1.3	0.6	0.4	2.3		0.9	0.4	0.2	1.6	
**RSH GGA**	ωB97	1.5	1.5	0.0	5.2		0.8	0.6	0.0	2.1	
	ωB97X	3.4	2.1	1.5	8.3		2.0	0.8	0.9	3.4	
	ωB97X-D	2.4	1.4	0.5	5.0		1.5	0.6	0.3	2.4	
	ωB97X-D3	4.9	1.4	3.0	7.0		3.3	1.5	1.5	5.8	P(2B,2NB)
	ωB97X-V	0.9	0.6	0.1	2.1		0.6	0.4	0.0	1.3	
	ωB97X-D4	2.2	0.8	1.3	4.2		1.5	0.7	0.5	2.8	
	HSE06	1.8	1.3	0.0	4.6		1.2	0.7	0.0	2.3	
	HSE06-D3	3.4	1.4	1.8	6.7		2.2	0.8	1.1	3.2	
	LC-PBE	3.6	2.1	0.9	6.9		2.3	1.3	0.6	4.8	
	LC-ωPBE	1.2	1.2	0.2	3.7		0.9	0.8	0.1	2.2	
	LC-ωPBE-D3	3.2	1.2	1.9	6.2	A(N3)	2.1	0.9	1.0	3.3	
	CAM-B3LYP	4.7	1.7	2.2	7.1		3.0	0.8	1.7	4.1	
	CAM-B3LYP-D3	6.6	2.0	3.1	9.4	P(1B1NB,2B,2NB), A(N3), T/U(O4)	4.1	0.9	2.8	5.5	
**GH meta-GGA**	M06	2.4	2.0	0.4	5.6		1.4	0.9	0.2	2.8	
	M06-2X	2.3	0.7	1.2	3.7	P(2B), A(N3)	1.5	0.5	0.9	2.8	
	M06-HF	6.7	2.1	2.7	9.5	P(1B1NB,2B)	4.2	1.1	3.1	7.0	
	MN15	1.6	1.4	0.1	4.1		0.8	0.6	0.1	1.9	
	BMK	3.0	2.3	0.7	7.5		1.7	0.9	0.5	3.3	
	PW6B95	2.4	1.1	0.9	4.4		1.6	0.7	0.4	2.6	
**GH GGA**	BH&HLYP	5.3	2.2	1.9	8.4		3.3	1.0	1.2	4.7	

	SOGGA11-X	1.9	1.0	0.1	3.6		1.2	0.6	0.0	2.0	
	B3PW91	1.4	1.3	0.2	3.7		0.7	0.6	0.1	1.9	
	B3PW91-D3	2.3	1.3	0.9	5.5	A(N3)	1.6	0.9	0.4	3.3	
	PBE0	1.8	1.3	0.1	4.5		1.2	0.7	0.0	2.2	
	PBE0-D3	3.4	1.4	2.0	6.5		2.2	0.8	1.2	3.2	
	B3LYP	2.4	1.4	0.7	4.9		1.6	0.8	0.4	2.7	
	B3LYP-D3	5.6	1.7	2.6	8.7		3.6	1.1	2.6	6.1	P(2B)
	B3LYP-D4	3.5	1.6	1.6	6.5		2.2	0.9	1.0	3.6	
	X3LYP	3.7	1.5	1.6	6.3		2.3	0.8	1.2	3.5	
	X3LYP-D3	7.3	2.0	3.6	10.6		4.7	1.2	3.4	7.5	P(2B)
	O3LYP	1.0	0.9	0.1	3.3	A(N3)	0.6	0.4	0.1	1.5	
	O3LYP-D3	5.1	1.8	3.0	8.7		3.4	1.4	1.4	6.4	P(2B)
	B97-2	1.4	1.3	0.0	3.7		0.8	0.5	0.0	1.9	
	TPSSh	1.4	1.1	0.1	3.9		1.0	0.7	0.0	2.4	
**Local meta-GGA**	revTPSS	1.1	0.8	0.0	2.7		0.8	0.6	0.0	2.4	P(2B)
	TPSS	1.3	1.0	0.0	3.6	A(N3)	0.9	0.8	0.0	2.9	P(2B)
	TPSS-D3	3.2	1.7	1.2	6.5		2.2	1.3	0.6	5.3	P(2B)
	TPSS-D4	1.8	1.5	0.0	5.1		1.3	1.1	0.0	3.7	
	M06-L	3.3	2.4	0.8	7.6		1.9	1.0	0.4	3.9	
	M11-L	3.5	1.9	1.0	6.4		2.3	1.4	0.4	5.5	
	MN12-L	1.3	0.7	0.1	2.1		0.9	0.6	0.2	2.5	P(2B)
	MN15-L	3.3	1.6	0.7	5.6		1.9	0.6	1.2	3.1	
**Local GGA**	mPW91	1.1	1.1	0.0	3.5		0.7	0.6	0.0	1.6	
	BLYP	1.2	1.0	0.0	3.2		0.8	0.9	0.0	3.3	
	BLYP-D3	4.4	1.9	1.8	7.8		2.9	1.6	1.2	7.3	P(2B)
	BP86	3.6	2.2	0.2	7.2		2.1	1.0	0.3	3.7	
	BP86-D3	1.4	1.0	0.1	3.0		1.0	0.8	0.1	3.2	P(2B)
	PBE	1.3	0.9	0.0	3.6	A(N3)	0.8	0.6	0.0	2.4	P(2B)
	PBE-D3	2.0	1.7	0.0	5.6		1.4	1.2	0.0	4.2	P(2B)
	PBE-D4	1.6	1.2	0.2	4.7		1.0	0.8	0.1	3.0	P(2B)
**Local LDA**	SVWN5	5.0	2.7	1.4	10.1		3.2	1.5	0.7	5.6	

[a]Complexes resulting in an outlier in the boxplot statistics are listed according to the nucleic acid component (A, C, T, G, U, or P) and binding site (in parentheses). See Figure 2 for definitions of the complexes considered in present work and Figure 4 for the outliers.

Table S4. MPEs (%) and MUEs (kcal/mol) in DFT binding strengths along with corresponding standard (SD), minimum (Min), and maximum (Max) deviations evaluated across Na^{+}–nucleic acid complexes for each functional relative to CCSD(T)/CBS reference values, as well as outliers based on the boxplot analysis.

Family	Functional	MPE	SD	Min	Max	Outliers[a]	MUE	SD	Min	MAX	Outlier[a]
**Double-Hybrid**	B2-PLYP	3.5	2.1	1.5	7.9	A(N3), G(N3)	1.6	0.6	0.6	2.5	
	B2-PLYP-D3	1.1	0.9	0.0	2.9		0.4	0.3	0.0	0.9	
	mPW2-PLYP	1.6	1.6	0.5	5.3	A(N3,N7), G(N3)	0.7	0.5	0.2	1.5	
	PBE-QIDH	4.9	3.2	1.4	12.7	A(N3,N7)	2.1	0.7	1.1	3.3	
	PBE0-DH	3.4	3.2	0.6	11.2	A(N7), G(N3)	1.4	0.8	0.3	2.9	G(N3)
	DSD-PBEP86	3.6	1.7	0.9	6.5	P(1B1NB,2B,2NB)	1.6	0.4	1.1	2.2	
**RSH**	M11	2.2	3.0	0.1	9.4	A(N1,N7), G(N3)	0.8	0.9	0.0	2.5	A(N1,N7), G(N3)
	MN12-SX	4.3	3.4	1.3	12.5	A(N7), G(N3)	1.9	0.9	0.5	3.3	
	MN12-SX-D3	2.7	2.7	0.2	9.4	A(N1,N7), G(N3)	1.1	0.8	0.1	2.5	
**RSH meta-GGA**	ωB97M-V	1.2	1.5	0.0	5.2	G(N3)	0.4	0.4	0.0	1.3	A(N7), G(N3)
**RSH GGA**	ωB97	3.5	3.1	1.2	11.6	A(N1,N7), G(N3)	1.5	0.7	0.5	3.0	G(N3)
	ωB97X	5.5	4.1	2.0	15.7	A(N1,N7), G(N3)	2.4	1.0	1.1	4.1	
	ωB97X-D	5.3	3.2	2.4	13.2	G(N3)	2.5	0.8	1.1	3.4	
	ωB97X-D3	3.6	1.1	2.0	5.7	U(O2), A(N3,N7)	2.1	1.5	0.7	5.2	P(1B1NB,2B,2NB)
	ωB97X-V	1.8	2.0	0.3	7.3	G(N3)	0.7	0.5	0.1	1.9	A(N1,N7), G(N3)
	ωB97X-D4	1.5	0.7	0.8	2.8		0.9	0.6	0.3	1.9	P(2B)
	HSE06	2.3	2.7	0.1	8.2	A(N7), G(N3)	1.0	0.8	0.0	2.4	
	HSE06-D3	1.6	1.3	0.1	3.4		0.7	0.3	0.0	1.1	
	LC-PBE	2.7	3.0	0.3	10.8	G(N3)	1.1	0.8	0.1	2.8	
	LC-ωPBE	3.8	3.5	0.8	12.5	A(N7), G(N3)	1.5	0.9	0.7	3.3	A(N7), G(N3)
	LC-ωPBE-D3	1.6	1.8	0.3	6.7	A(N1,N7), G(N3)	0.7	0.4	0.3	1.7	G(N3)
	CAM-B3LYP	2.4	1.3	0.4	4.4	U(O4)	1.0	0.3	0.5	1.6	
	CAM-B3LYP-D3	4.1	2.1	1.3	7.2		2.0	0.8	0.3	2.9	G(N3)
**GH meta-GGA**	M06	7.9	5.0	2.9	18.9	A(N7), G(N3)	3.5	1.1	2.0	5.4	
	M06-2X	1.1	1.4	0.0	4.8	A(N1,N7),G(N3)	0.4	0.4	0.1	1.2	
	M06-HF	2.6	0.8	1.4	3.8		1.4	0.8	0.5	3.7	P(2B)
	MN15	5.2	3.7	1.5	13.7	G(N3)	2.2	0.8	1.2	3.8	A(N7), G(N3)
	BMK	6.7	4.9	1.9	17.8	G(N3)	2.9	1.1	1.3	4.8	
	PW6B95	2.0	2.5	0.1	7.3	A(N1,N7),G(N3)	0.7	0.7	0.1	2.2	A(N1,N7), G(N3)
**GH GGA**	BH&HLYP	2.8	2.2	0.1	8.5	G(N3)	1.2	0.6	0.1	2.2	

	SOGGA11-X	3.1	3.0	0.4	11.2	G(N3)	1.3	0.8	0.1	2.9	
	B3PW91	6.3	4.0	2.2	14.9	A(N7), G(N3)	2.8	1.0	1.4	4.4	
	B3PW91-D3	1.8	1.7	0.1	5.4	G(N3)	0.7	0.5	0.1	1.4	
	PBE0	2.4	2.7	0.0	8.4	A(N7), G(N3)	1.0	0.8	0.0	2.4	
	PBE0-D3	1.5	1.2	0.1	3.8		0.6	0.3	0.0	1.0	
	B3LYP	2.1	2.3	0.1	7.0	A(N1,N7), G(N3)	0.9	0.7	0.0	2.2	
	B3LYP-D3	3.0	1.8	1.3	6.3	A(N3)	1.5	0.6	0.5	2.3	P(2B)
	B3LYP-D4	1.9	1.5	0.3	4.4	A(N3), T(O4), U(O4)	0.8	0.4	0.4	1.6	T(O4)
	X3LYP	1.7	1.5	0.1	4.4		0.6	0.4	0.1	1.4	
	X3LYP-D3	6.0	2.1	2.9	9.9		2.9	0.9	1.9	4.6	
	O3LYP	5.6	3.2	2.3	11.6		2.6	1.0	1.0	3.9	
	O3LYP-D3	2.6	1.9	0.1	7.3	A(N3)	1.5	1.1	0.1	3.9	
	B97-2	6.5	4.0	2.4	15.4	A(N7), G(N3)	2.9	1.0	1.5	4.4	
	TPSSh	3.5	2.5	1.1	7.9		1.6	0.7	0.5	2.8	A(N3,N7), U(O4)
**Local meta-GGA**	revTPSS	4.0	2.2	1.1	7.8		1.8	0.6	0.6	2.8	
	TPSS	3.7	2.3	0.9	8.0		1.7	0.7	0.3	2.9	A(N3)
	TPSS-D3	1.4	1.0	0.0	3.6	A(N3)	0.6	0.3	0.0	1.1	
	TPSS-D4	1.8	1.4	0.0	4.7		0.8	0.5	0.0	1.7	
	M06-L	8.0	5.0	2.6	18.0	A(N7), G(N3)	3.5	1.1	1.9	5.6	
	M11-L	7.4	4.3	2.0	16.2		3.3	0.9	1.8	4.7	
	MN12-L	5.9	4.4	1.7	16.5	A(N7), G(N3)	2.5	1.0	1.0	4.3	
	MN15-L	5.7	3.1	1.8	12.5		2.5	0.6	1.5	3.3	A(N7), T(O4), U(O4)
**Local GGA**	mPW91	4.0	3.4	1.2	11.6	A(N1), G(N3)	1.8	0.9	0.6	3.3	
	BLYP	3.2	2.1	0.2	7.4		1.6	0.9	0.1	3.1	
	BLYP-D3	2.5	2.2	0.3	7.6	A(N3)	1.1	0.7	0.1	2.4	
	BP86	7.4	3.5	3.0	13.3		3.5	1.1	1.6	4.9	
	BP86-D3	2.6	1.7	0.1	4.7		1.1	0.6	0.1	1.9	
	PBE	3.0	1.9	1.0	6.9		1.4	0.7	0.4	2.5	
	PBE-D3	1.5	1.3	0.2	4.4		0.6	0.4	0.1	1.4	
	PBE-D4	1.7	1.4	0.2	4.4		0.8	0.5	0.2	1.6	
**Local LDA**	SVWN5	4.4	3.0	0.4	10.5		2.2	1.2	0.1	4.0	

[a]Complexes resulting in an outlier in the boxplot statistics are listed according to the nucleic acid component (A, C, T, G, U, or P) and binding site (in parentheses). See Figure 2 for definitions of the complexes considered in present work and Figure 4 for the outliers.

Table S5. MPEs (%) and MUEs (kcal/mol) in DFT binding strengths along with corresponding standard (SD), minimum (Min), and maximum (Max) deviations evaluated across K^+–nucleic acid complexes for each functional relative to CCSD(T)/CBS reference values, as well as outliers based on the boxplot analysis.

Family	Functional	MPE	SD	Min	Max	Outliers[a]	MUE	SD	Min	MAX	Outliers[a]
**Double-Hybrid**	B2-PLYP	1.1	0.9	0.2	2.9		0.3	0.2	0.1	0.6	P(2NB)
	B2-PLYP-D3	4.4	3.1	1.2	11.7		1.2	0.5	0.5	2.3	P(2B)
	mPW2-PLYP	1.2	1.3	0.2	4.6	G(N3)	0.3	0.1	0.1	0.5	
	PBE-QIDH	1.1	0.8	0.1	3.1	G(N3)	0.3	0.2	0.1	0.7	G(N7)
	PBE0-DH	1.6	1.2	0.1	4.6	G(N3)	0.4	0.2	0.1	1.0	G(N7)
	DSD-PBEP86	3.0	2.8	0.5	9.7		0.9	0.6	0.1	2.1	P(2B)
**RSH**	M11	2.8	2.2	0.0	5.6		1.0	0.6	0.0	2.1	G(N7)
	MN12-SX	2.8	1.9	0.6	6.5		0.9	0.6	0.1	2.1	
	MN12-SX-D3	6.6	3.1	2.1	11.5		2.0	0.7	1.1	3.5	P(2NB)
**RSH meta-GGA**	ωB97M-V	1.0	1.2	0.2	4.5	G(N3)	0.3	0.2	0.0	0.5	
**RSH GGA**	ωB97	1.2	1.0	0.0	4.1	G(N3)	0.3	0.2	0.0	0.6	
	ωB97X	1.4	1.2	0.0	4.4	G(N3)	0.3	0.2	0.0	0.8	G(N7)
	ωB97X-D	5.7	3.8	1.1	13.3	G(N3)	1.5	0.5	0.6	2.6	
	ωB97X-D3	12.2	7.2	5.3	30.0	G(N3)	3.8	1.9	1.8	7.8	P(2B,2NB)
	ωB97X-V	1.1	1.0	0.1	4.0	G(N3)	0.3	0.1	0.1	0.5	
	ωB97X-D4	9.6	5.2	3.4	22.2	G(N3)	2.9	1.0	1.6	4.8	
	HSE06	1.9	1.5	0.1	5.1		0.4	0.2	0.1	0.8	
	HSE06-D3	4.9	2.9	1.4	10.4		1.4	0.5	0.6	2.2	
	LC-PBE	2.7	2.4	0.2	7.9		0.7	0.5	0.0	1.9	G(N7)
	LC-ωPBE	1.7	1.5	0.0	4.7		0.4	0.3	0.1	1.1	
	LC-ωPBE-D3	5.8	3.1	2.0	12.2	A(N3), G(N3)	1.8	0.6	0.9	2.8	
	CAM-B3LYP	2.1	2.2	0.1	7.6	G(N3)	0.5	0.3	0.0	0.9	
	CAM-B3LYP-D3	5.4	2.5	1.6	9.7	A(N3), P(2NB)	1.6	0.5	1.0	2.4	
**GH meta-GGA**	M06	2.0	1.5	0.1	5.7	A(N3)	0.5	0.3	0.2	1.0	
	M06-2X	0.9	0.7	0.0	2.2		0.3	0.3	0.0	0.8	
	M06-HF	2.0	1.3	0.3	4.0		0.7	0.6	0.1	2.0	
	MN15	1.7	1.8	0.1	6.3	A(N7), G(N3)	0.4	0.2	0.1	0.8	
	BMK	1.7	1.5	0.2	4.3		0.5	0.4	0.0	1.3	
	PW6B95	1.8	2.0	0.1	6.7	G(N3)	0.4	0.3	0.1	0.9	
**GH GGA**	BH&HLYP	3.0	2.4	0.2	9.3	G(N3)	0.8	0.4	0.2	1.6	
	SOGGA11-X	1.4	1.3	0.1	4.0		0.4	0.3	0.0	0.9	

	B3PW91	1.7	1.3	0.0	4.4		0.4	0.3	0.0	0.9	
	B3PW91-D3	9.5	5.9	3.0	23.6	G(N3)	2.7	0.8	1.4	4.5	
	PBE0	1.8	1.4	0.1	4.8		0.4	0.2	0.1	0.8	
	PBE0-D3	4.3	2.5	1.3	9.2		1.3	0.5	0.5	2.1	
	B3LYP	1.8	1.7	0.1	5.4		0.4	0.2	0.0	0.8	
	B3LYP-D3	8.4	4.8	2.5	19.4	G(N3)	2.4	0.7	1.4	4.0	P(2B)
	B3LYP-D4	5.5	3.0	1.3	10.5		1.5	0.4	1.0	2.2	
	X3LYP	1.9	1.9	0.2	6.3		0.5	0.3	0.0	0.9	
	X3LYP-D3	11.6	6.9	4.0	28.3		3.4	1.2	1.8	6.0	P(2B)
	O3LYP	2.1	1.8	0.1	4.9		0.6	0.3	0.0	0.9	T(O4)
	O3LYP-D3	15.5	11.0	6.2	43.6		4.6	2.1	1.8	8.7	P(2B)
	B97-2	1.6	1.3	0.0	3.8		0.4	0.2	0.1	0.7	
	TPSSh	1.4	1.1	0.0	4.2	A(N3)	0.4	0.2	0.0	0.8	A(N3), C(O2), G(N3), P(1B1NB)
**Local meta-GGA**	revTPSS	1.5	1.4	0.1	4.0		0.4	0.3	0.1	1.1	
	TPSS	1.6	1.4	0.1	4.5		0.4	0.3	0.0	1.0	
	TPSS-D3	5.8	4.8	1.1	17.8	A(N3), G(N3)	1.6	0.8	0.5	3.3	P(2B)
	TPSS-D4	4.3	3.7	0.6	13.1	G(N3)	1.1	0.6	0.4	2.3	
	M06-L	2.0	2.0	0.5	8.1	A(N3)	0.6	0.5	0.1	1.6	P(2B), A(N3)
	M11-L	2.9	2.2	0.4	9.4	A(N3)	0.9	0.6	0.1	2.4	P(2NB)
	MN12-L	3.2	2.4	0.5	8.4		1.0	0.7	0.1	2.2	
	MN15-L	1.3	0.8	0.2	2.5		0.4	0.3	0.1	1.2	P(1B1NB,2B)
**Local GGA**	mPW91	1.8	1.4	0.0	4.5		0.4	0.2	0.0	0.9	
	BLYP	2.3	1.7	0.3	5.5		0.7	0.5	0.1	1.9	
	BLYP-D3	8.7	6.9	1.7	26.3	G(N3)	2.3	0.9	1.1	4.2	
	BP86	1.8	1.7	0.1	4.8		0.6	0.4	0.0	1.4	
	BP86-D3	8.9	6.9	2.1	25.1		2.4	0.9	0.9	4.4	A(N3)
	PBE	2.7	1.9	0.6	6.0		0.8	0.5	0.2	1.9	
	PBE-D3	2.6	3.0	0.3	9.3	A(N3), G(N3)	0.6	0.5	0.2	1.6	
	PBE-D4	2.1	2.6	0.0	8.0	A(N3), G(N3)	0.5	0.4	0.0	1.4	
**Local LDA**	SVWN5	3.6	3.4	0.3	10.5		0.9	0.6	0.1	2.0	

[a]Complexes resulting in an outlier in the boxplot statistics are listed according to the nucleic acid component (A, C, T, G, U, or P) and binding site (in parentheses). See Figure 2 for definitions of the complexes considered in present work and Figure 5 for the outliers.

Table S6. MPEs (%) and MUEs (kcal/mol) in DFT binding strengths along with corresponding standard (SD), minimum (Min), and maximum (Max) deviations evaluated across Rb^{+}–nucleic acid complexes for each functional relative to CCSD(T)/CBS reference values, as well as outliers based on the boxplot analysis.

Family	Functional	MPE	SD	Min	Max	Outliers[a]	MUE	SD	Min	MAX	Outliers[a]
**Double-Hybrid**	B2-PLYP	0.9	0.7	0.0	2.2		0.2	0.1	0.0	0.5	
	B2-PLYP-D3	5.5	3.2	1.5	11.1		1.6	0.5	0.7	2.7	P(2B)
	mPW2-PLYP	1.0	0.7	0.0	1.9		0.2	0.1	0.0	0.4	
	PBE-QIDH	1.2	0.9	0.3	2.5		0.4	0.2	0.1	0.8	
	PBE0-DH	1.4	1.4	0.0	3.5		0.4	0.4	0.0	1.1	
	DSD-PBEP86	3.7	2.6	1.4	8.7	A(N7)	1.1	0.6	0.3	2.5	
**RSH**	M11	3.9	2.5	0.5	6.8		1.2	0.6	0.1	2.2	
	MN12-SX	4.1	2.2	1.2	8.1		1.2	0.5	0.6	2.3	
	MN12-SX-D3	8.5	4.0	2.9	15.0		2.5	0.8	1.3	4.0	
**RSH meta-GGA**	ωB97M-V	0.8	0.5	0.0	1.7		0.2	0.1	0.0	0.4	
**RSH GGA**	ωB97	1.1	1.1	0.1	2.8		0.3	0.3	0.0	0.9	
	ωB97X	1.3	1.3	0.0	3.0		0.3	0.4	0.0	1.0	
	ωB97X-D	7.3	4.6	1.6	14.8		1.9	0.6	0.9	3.0	P(2B)
	ωB97X-D3	13.7	6.9	6.2	28.1	A(N1,N7)	4.4	2.2	2.1	8.8	
	ωB97X-V	0.9	0.9	0.0	2.0		0.2	0.2	0.0	0.7	
	ωB97X-D4	11.7	5.6	4.5	22.2	A(N7)	3.6	1.4	2.0	6.2	
	HSE06	1.7	1.4	0.0	4.5		0.4	0.3	0.0	1.0	
	HSE06-D3	6.2	3.4	1.8	13.0		1.7	0.5	0.8	2.5	
	LC-PBE	2.7	2.0	0.4	5.5		0.8	0.6	0.2	2.0	G(N7), C(O2)
	LC-ωPBE	1.6	1.4	0.1	3.9		0.4	0.4	0.0	1.2	A(N3), C(O2)
	LC-ωPBE-D3	6.9	3.4	2.3	13.2		2.0	0.7	1.1	3.1	
	CAM-B3LYP	2.0	1.4	0.0	3.6		0.5	0.3	0.0	1.1	
	CAM-B3LYP-D3	6.9	3.3	2.0	12.4		2.0	0.5	1.3	2.7	
**GH meta-GGA**	M06	2.3	1.9	0.6	7.1	A(N3)	0.7	0.4	0.1	1.2	
	M06-2X	1.6	0.9	0.6	3.0		0.5	0.3	0.2	0.9	
	M06-HF	2.8	1.5	0.7	5.1		0.9	0.5	0.1	2.0	P(2NB)
	MN15	1.5	1.2	0.1	3.0		0.4	0.2	0.0	0.7	
	BMK	2.6	1.8	0.7	5.4		0.8	0.4	0.2	1.5	
	PW6B95	1.7	1.3	0.0	3.6		0.4	0.2	0.0	0.7	
**GH GGA**	BH&HLYP	2.9	2.0	0.3	5.8		0.9	0.5	0.2	1.8	
	SOGGA11-X	2.0	1.3	0.3	3.9		0.6	0.3	0.2	1.1	

	B3PW91	1.7	1.7	0.0	5.5		0.4	0.3	0.0	1.1	
	B3PW91-D3	10.5	5.6	3.4	19.6		3.0	0.9	1.6	4.8	
	PBE0	1.7	1.3	0.0	4.3		0.4	0.3	0.0	0.9	
	PBE0-D3	5.3	3.0	1.6	11.2		1.5	0.5	0.6	2.3	
	B3LYP	1.8	1.5	0.0	4.2		0.4	0.3	0.0	0.8	
	B3LYP-D3	9.5	4.9	2.9	17.3		2.7	0.8	1.6	4.4	P(2B)
	B3LYP-D4	6.9	3.5	1.7	13.0		1.9	0.5	1.2	2.7	
	X3LYP	1.8	1.5	0.1	3.7		0.4	0.3	0.0	0.8	
	X3LYP-D3	13.7	7.3	5.0	27.3		4.1	1.5	2.1	7.0	
	O3LYP	1.8	1.2	0.5	3.6		0.5	0.2	0.2	0.7	
	O3LYP-D3	18.6	11.4	8.1	42.1		5.7	2.7	2.3	10.7	
	B97-2	1.6	1.4	0.0	4.5		0.4	0.3	0.0	0.9	
	TPSSh	1.5	1.4	0.2	5.1	A(N3)	0.4	0.2	0.1	0.8	
**Local meta-GGA**	revTPSS	1.5	1.4	0.2	5.0	A(N3)	0.4	0.3	0.1	1.0	
	TPSS	1.6	1.4	0.0	5.4	A(N3)	0.4	0.2	0.0	0.9	A(N3), P(1B1NB)
	TPSS-D3	6.3	4.1	1.3	14.7		1.8	0.7	0.7	3.5	P(2B)
	TPSS-D4	5.1	3.4	0.8	12.6		1.4	0.6	0.6	2.6	
	M06-L	2.4	2.7	0.1	9.8	A(N3)	0.8	0.6	0.0	1.8	
	M11-L	3.8	2.6	1.3	10.9	A(N3)	1.2	0.6	0.4	2.5	
	MN12-L	4.3	2.7	1.3	10.0		1.3	0.7	0.5	2.3	
	MN15-L	1.8	1.3	0.0	3.6		0.4	0.2	0.0	0.7	
**Local GGA**	mPW91	1.7	1.5	0.1	4.6		0.4	0.3	0.0	1.0	
	BLYP	2.1	1.5	0.1	4.6		0.6	0.4	0.0	1.7	P(2NB)
	BLYP-D3	9.4	5.9	2.1	19.8		2.6	0.9	1.4	4.6	P(2B)
	BP86	1.8	1.6	0.2	6.0	A(N3)	0.5	0.3	0.2	1.2	A(N3), P(2NB)
	BP86-D3	10.2	6.8	2.6	22.1		2.8	1.0	1.3	4.9	
	PBE	2.3	1.8	0.1	4.7		0.7	0.5	0.0	1.7	P(2NB)
	PBE-D3	2.8	3.0	0.1	10.6	A(N3)	0.7	0.6	0.1	1.7	
	PBE-D4	2.5	2.8	0.2	10.0	A(N3)	0.6	0.5	0.0	1.6	
**Local LDA**	SVWN5	3.3	2.9	0.2	9.1		0.8	0.5	0.1	1.5	

[a]Complexes resulting in an outlier in the boxplot statistics are listed according to the nucleic acid component (A, C, T, G, U, or P) and binding site (in parentheses). See Figure 2 for definitions of the complexes considered in present work and Figure 6 for the outliers.

Table S7. MPEs (%) and MUEs (kcal/mol) in DFT binding strengths along with corresponding standard (SD), minimum (Min), and maximum (Max) deviations evaluated across Cs^{+}–nucleic acid complexes for each functional relative to CCSD(T)/CBS reference values, as well as outliers based on the boxplot analysis.

Family	Functional	MPE	SD	Min	Max	Outliers[a]	MUE	SD	Min	MAX	Outliers[a]
**Double-Hybrid**	B2-PLYP	1.0	0.7	0.0	2.1		0.2	0.1	0.0	0.5	P(2NB)
	B2-PLYP-D3	6.1	4.0	1.8	13.9		1.6	0.6	0.7	2.8	
	mPW2-PLYP	1.1	0.8	0.0	2.4		0.2	0.1	0.0	0.4	
	PBE-QIDH	1.1	0.8	0.1	2.3		0.3	0.2	0.0	0.6	A(N1), G(N7), C(O2)
	PBE0-DH	1.5	1.1	0.3	3.5		0.4	0.2	0.1	0.9	
	DSD-PBEP86	3.6	2.9	1.0	9.5	A(N7)	1.0	0.6	0.2	2.3	
**RSH**	M11	4.0	2.5	0.7	7.7		1.2	0.6	0.1	2.1	
	MN12-SX	5.0	2.4	2.0	10.1		1.5	0.9	0.6	3.4	
	MN12-SX-D3	10.8	5.3	4.3	19.4		3.0	1.2	1.5	5.5	
**RSH meta-GGA**	ωB97M-V	0.9	0.6	0.1	1.9		0.2	0.1	0.1	0.3	
**RSH GGA**	ωB97	1.2	0.7	0.1	2.3		0.3	0.2	0.1	0.6	
	ωB97X	1.4	1.0	0.1	2.7		0.3	0.2	0.1	0.8	G(N7), C(O2)
	ωB97X-D	18.8	13.9	4.0	47.2		4.4	1.3	2.3	6.7	
	ωB97X-D3	15.1	8.2	6.9	33.2	A(N1,N7)	4.5	2.3	2.1	9.0	
	ωB97X-V	1.0	0.5	0.1	1.7		0.2	0.1	0.1	0.4	
	ωB97X-D4	14.4	7.5	5.6	29.1	A(N7)	4.0	1.7	2.1	7.1	
	HSE06	1.8	1.4	0.1	4.8		0.4	0.2	0.1	0.8	
	HSE06-D3	7.1	4.2	2.1	15.2		1.8	0.6	0.8	2.8	
	LC-PBE	2.4	2.1	0.1	5.0		0.6	0.5	0.0	1.7	G(N7), C(O2)
	LC-ωPBE	1.7	1.3	0.0	3.7		0.4	0.3	0.0	0.9	
	LC-ωPBE-D3	7.4	4.2	2.4	14.9		2.0	0.6	0.9	3.0	
	CAM-B3LYP	1.9	1.7	0.0	4.5		0.4	0.3	0.0	0.9	
	CAM-B3LYP-D3	7.6	3.9	2.2	14.5		2.0	0.5	1.2	2.9	
**GH meta-GGA**	M06	2.6	2.1	0.8	8.6	A(N3)	0.8	0.5	0.1	1.8	
	M06-2X	1.5	1.0	0.5	3.4		0.4	0.3	0.1	1.0	
	M06-HF	2.5	1.5	0.3	4.7		0.7	0.5	0.1	1.9	P(2NB)
	MN15	1.5	1.3	0.1	4.1		0.3	0.2	0.1	0.6	
	BMK	2.3	1.9	0.4	5.7		0.6	0.4	0.1	1.3	
	PW6B95	1.8	1.4	0.1	4.2		0.4	0.2	0.1	0.6	
**GH GGA**	BH&HLYP	2.8	1.7	0.4	5.4		0.8	0.4	0.3	1.5	
	SOGGA11-X	1.8	1.3	0.4	3.7		0.5	0.3	0.1	0.9	

	B3PW91	1.9	1.6	0.3	5.9		0.4	0.2	0.1	0.9	
	B3PW91-D3	11.8	6.9	3.9	24.1		3.1	1.0	1.5	5.1	P(2B)
	PBE0	1.7	1.3	0.0	4.5	A(N3)	0.4	0.2	0.0	0.8	G(N7)
	PBE0-D3	5.8	3.5	1.9	12.7		1.5	0.6	0.6	2.5	
	B3LYP	1.8	1.7	0.0	4.5		0.4	0.3	0.0	0.7	
	B3LYP-D3	10.6	5.9	3.4	20.7		2.8	0.9	1.5	4.7	P(2B)
	B3LYP-D4	8.2	4.2	2.4	16.0		2.2	0.6	1.3	3.2	
	X3LYP	1.8	1.7	0.1	4.4		0.4	0.3	0.0	0.7	
	X3LYP-D3	16.6	9.9	6.2	36.8	A(N7)	4.5	1.8	2.2	7.9	
	O3LYP	1.9	1.5	0.2	4.0		0.4	0.2	0.1	0.6	
	O3LYP-D3	23.9	16.1	10.2	59.0		6.7	3.4	2.7	12.7	
	B97-2	1.7	1.3	0.2	4.7	A(N3)	0.4	0.2	0.2	0.7	
	TPSSh	1.6	1.5	0.0	5.5	A(N3)	0.4	0.2	0.0	0.8	
**Local meta-GGA**	revTPSS	1.6	1.6	0.1	5.4	A(N3)	0.4	0.2	0.1	0.8	
	TPSS	1.8	1.5	0.4	5.9	A(N3)	0.4	0.2	0.2	0.8	A(N3)
	TPSS-D3	7.1	4.8	1.9	16.7		1.9	0.9	0.7	3.9	P(2B)
	TPSS-D4	6.3	4.1	1.6	15.1		1.7	0.7	0.7	3.3	P(2B)
	M06-L	2.9	3.0	0.3	11.3	A(N3)	1.0	0.8	0.0	2.6	
	M11-L	4.5	3.0	1.9	12.9	A(N3)	1.4	1.0	0.4	3.7	P(2NB)
	MN12-L	4.9	2.9	1.9	11.7		1.5	0.9	0.5	3.3	
	MN15-L	1.8	1.4	0.1	3.9		0.4	0.1	0.0	0.6	C(O2)
**Local GGA**	mPW91	1.8	1.3	0.2	4.8		0.4	0.2	0.2	0.9	
	BLYP	2.2	1.9	0.1	5.4		0.5	0.3	0.1	1.2	
	BLYP-D3	10.8	7.0	2.9	23.0		2.8	1.1	1.4	5.2	P(2B)
	BP86	1.9	1.8	0.1	6.7	A(N3)	0.4	0.2	0.1	0.9	A(N3)
	BP86-D3	12.1	8.5	3.4	27.5		3.1	1.2	1.4	5.5	
	PBE	2.4	2.1	0.1	5.0		0.6	0.4	0.1	1.3	
	PBE-D3	3.2	3.4	0.0	11.9	A(N3)	0.8	0.6	0.0	2.0	
	PBE-D4	3.3	3.4	0.0	11.9	A(N3)	0.8	0.6	0.0	1.8	
**Local LDA**	SVWN5	3.3	3.1	0.0	9.1		0.7	0.4	0.0	1.3	

[a]Complexes resulting in an outlier in the boxplot statistics are listed according to the nucleic acid component (A, C, T, G, U, or P) and binding site (in parentheses). See Figure 2 for definitions of the complexes considered in present work and Figure 6 for the outliers.

Table S8. MPEs (%) and MUEs (kcal/mol) in DFT binding strengths along with corresponding standard (SD), minimum (Min), and maximum (Max) deviations evaluated across group I metal–nucleic acid complexes for each functional relative to CCSD(T)/CBS reference values, as well as outliers based on the boxplot analysis.

Family	Functional	MPE	SD	Min	Max	Outliers[a]	MUE	SD	Min	MAX	Outliers[a]
**Double-Hybrid**	B2-PLYP	1.5	1.5	0.0	7.9	Na(A(N1,N7), G(N3))	0.6	0.7	0.0	2.5	Na(A(N7), G(N3), P(2B,2NB))
	B2-PLYP-D3	3.9	3.2	0.0	13.9	KG(N3), RbA(N3,N7), CsA(N1,N3,N7)	1.3	0.7	0.0	3.7	LiP(2B)
	mPW2-PLYP	1.6	1.3	0.0	5.3	NaG(N3)	0.7	0.7	0.0	2.7	LiP(2B)
	PBE-QIDH	1.8	2.2	0.1	12.7	Na(A(N1,N3,N7),G(N3), U(O2))	0.7	0.8	0.0	3.3	Na((A(N1,N3,N7), C(O2), G(N3,N7), P(1B1NB,2B,2NB), T/U(O2))
	PBE0-DH	1.9	1.9	0.0	11.2	Na(A(N1,N7), G(N3)),	0.7	0.7	0.0	2.9	Na(A(N1,N7), G(N3))
	DSD-PBEP86	2.9	2.5	0.1	9.7	KG(N3), RbA(N7), CsA(N7)	1.0	0.6	0.1	2.5	
**RSH**	M11	3.0	2.4	0.0	9.4		1.2	0.8	0.0	3.3	
	MN12-SX	3.4	2.6	0.1	12.5	NaG(N3)	1.3	0.8	0.0	3.4	NaG(N3), CsP(2NB)
	MN12-SX-D3	5.8	4.9	0.1	19.4		1.8	1.2	0.1	5.5	CsP(2NB)
**RSH meta-GGA**	ωB97M-V	1.1	1.0	0.0	5.2	NaG(N3),KG(N3)	0.4	0.4	0.0	1.6	Li(G(N7), P(1B1NB,2B,2NB))
**RSH GGA**	ωB97	1.7	1.9	0.0	11.6	Na(A(N1,N7),G(N3)), LiG(N3)	0.7	0.7	0.0	3.0	LiG(N3), Na(A(N1,N7), G(N3))
	ωB97X	2.7	2.8	0.0	15.7	Na(A(N1,N7), G(N3)), LiG(N3)	1.1	1.1	0.0	4.1	
	ωB97X-D	7.6	8.6	0.5	47.2	Cs(A(N1,N3,N7),T(O4))	2.3	1.3	0.3	6.7	Cs(A(N1,N3), G(N7))
	ωB97X-D3	9.7	7.3	2.0	33.2	KG(N3), RbA(N1,N7), CsA(N1,N3,N7)	3.6	2.0	0.7	9.0	RbP(2B), CsP(2B,2NB)
	ωB97X-V	1.1	1.2	0.0	7.3	Na(A(N7),G(N3)), KG(N3)	0.4	0.4	0.0	1.9	Li(A(N1,N3,N7), P(2NB),C(O2))
	ωB97X-D4	7.6	6.9	0.8	29.1	CsA(N7)	2.5	1.6	0.3	7.1	CsP(2B,2NB)
	HSE06	1.9	1.7	0.0	8.2	Na(A(N7), G(N3))	0.7	0.6	0.0	2.4	Li(A(N1,N7), G(N3), P(2NB), U(O4)), Na(A(N1,N7), G(N3))
	HSE06-D3	4.5	3.3	0.1	15.2	CsA(N3)	1.6	0.8	0.0	3.2	
	LC-PBE	2.8	2.3	0.1	10.8	NaG(N3)	1.1	1.0	0.0	4.8	Li(C(O2), G(N7), P(2NB))
	LC-ωPBE	2.0	2.1	0.0	12.5	Na(A(N1,N7), G(N3)),	0.7	0.7	0.0	3.3	LiG(N7), Na(A(N1,N7), G(N3))
	LC-ωPBE-D3	4.9	3.6	0.3	14.9		1.7	0.9	0.3	3.3	

	CAM-B3LYP	2.7	2.0	0.0	7.6		1.1	1.1	0.0	4.1	Li(C(O2), G(N7), P(1B1NB,2B,2NB), T/U(O4))
	CAM-B3LYP-D3	6.1	3.0	1.3	14.5	CsA(N3)	2.4	1.2	0.3	5.5	Li(G(N7),P(1B1NB,2B,2NB))
**GH meta-GGA**	M06	3.4	3.5	0.1	18.9	Na(A(N1,N7),G(N3))	1.4	1.3	0.1	5.4	Na(A(N1,N3), G(N3))
	M06-2X	1.5	1.1	0.0	4.8		0.7	0.6	0.0	2.8	LiP(1B1NB,2NB)
	M06-HF	3.4	2.3	0.3	9.5	Li(A(N1,N7), G(N3))	1.7	1.6	0.1	7.0	Li(C(O2), P(1B1NB,2NB))
	MN15	2.3	2.5	0.1	13.7	Na(A(N1,N7), G(N3))	0.8	0.9	0.0	3.8	Na(A(N1,N7), G(N3))
	BMK	3.3	3.2	0.2	17.8	Na(A(N1,N7), G(N3))	1.3	1.1	0.0	4.8	Na(A(N1,N7), G(N3))
	PW6B95	2.0	1.7	0.0	7.3	NaG(N3)	0.7	0.7	0.0	2.6	Li(A(N3), C(O2), G(N7), P(1B1NB,2B,2NB), T(O4))
**GH GGA**	BH&HLYP	3.4	2.3	0.1	9.3		1.4	1.2	0.1	4.7	Li(C(O2), G(N7), P(1B1NB,2B), T/U(O4)
	SOGGA11-X	2.0	1.8	0.1	11.2	Na(A(N7), G(N3))	0.8	0.6	0.0	2.9	Na(A(N7),G(N3))
	B3PW91	2.6	2.9	0.0	14.9	Na(A(N1,N7), G(N3)),	1.0	1.1	0.0	4.4	Na(A(N1,N7), G(N3), P(1B1NB,2B,2NB))
	B3PW91-D3	7.0	6.3	0.1	24.1	CsA(N3,N7), KG(N3)	2.2	1.3	0.1	5.1	
	PBE0	1.9	1.7	0.0	8.4	Na(A(N1,N7), G(N3))	0.7	0.6	0.0	2.4	Li(A(N3),G(N7)), NaG(N3)
	PBE0-D3	4.0	2.8	0.1	12.7	CsA(N3,N7)	1.4	0.8	0.0	3.2	LiG(N7),
	B3LYP	2.0	1.7	0.0	7.0		0.8	0.7	0.0	2.7	LiP(2B)
	B3LYP-D3	7.3	4.8	1.3	20.7	KG(N3), CsA(N3,N7)	2.6	1.1	0.5	6.1	LiP(1B1NB,2B)
	B3LYP-D4	5.1	3.6	0.3	16.0	CsA(N3)	1.7	0.8	0.4	3.6	
	X3LYP	2.2	1.8	0.1	6.3		0.9	0.9	0.0	3.5	Li(A(N3), P(1B1NB,2B), T/U(O4))
	X3LYP-D3	10.9	7.3	2.9	36.8	KG(N3), RbA(N3,N7), CsA(N1,N3,N7)	3.9	1.5	1.8	7.9	LiP(2B), CsP(2B)
	O3LYP	2.5	2.5	0.1	11.6	Na(A(N1,N7), G(N3))	0.9	1.0	0.0	3.9	Na(A(N1,N7), C(O2), G(N3,N7),P(1B1NB,2B,2NB))
	O3LYP-D3	12.8	12.6	0.1	59.0	KG(N3), RbA(N7), CsA(N1,N3,N7)	4.3	2.8	0.1	12.7	CsP(2B)
	B97-2	2.6	2.9	0.0	15.4	Na(A(N1,N7), G(N3))	1.0	1.1	0.0	4.4	Na(A(N1,N7), G(N3), P(1B1NB,2B,2NB))
	TPSSh	1.9	1.8	0.0	7.9	Na(A(N1), G(N3), U(O2)), RbA(N3), CsA(N3)	0.7	0.7	0.0	2.8	NaA(N7)
**Local meta-GGA**	revTPSS	1.9	1.8	0.0	7.8	Na(A(N1,N7), G(N3), U(O2))	0.8	0.7	0.0	2.8	LiP(2B), Na(A(N7), C(O2), P(1B1NB,2B))

	TPSS	2.0	1.8	0.0	8.0	Na(A(N7), G(N3), U(O2)), RbA(N3), CsA(N3)	0.8	0.7	0.0	2.9	LiP(1B1NB,2B), Na(A(N1,N7), C(O2), G(N7), P(1B1NB,2NB), U(O2)
	TPSS-D3	4.7	4.1	0.0	17.8	KG(N3), RbA(N3), CsA(N3,N7)	1.6	1.0	0.0	5.3	LiP(2B)
	TPSS-D4	3.8	3.4	0.0	15.1	K(A(N3), G(N3), RbA(N3), CsA(N3,N7)	1.2	0.8	0.0	3.7	LiP(2B), CsP(2B)
	M06-L	3.8	3.8	0.1	18.0	Na(A(N1,N7), G(N3)), CsA(N3)	1.6	1.4	0.0	5.6	NaA(N7)
	M11-L	4.4	3.2	0.4	16.2	Na(A(N7), G(N3), U(O2)), RbA(N3), CsA(N3)	1.9	1.3	0.1	5.5	
	MN12-L	3.9	3.2	0.1	16.5	Na(A(N7), G(N3)), CsA(N3)	1.4	1.0	0.1	4.3	Na(A(N7), G(N3))
	MN15-L	2.8	2.4	0.0	12.5	Na(A(N7), G(N3))	1.2	1.0	0.0	3.3	
**Local GGA**	mPW91	2.1	2.1	0.0	11.6	Na(A(N1,N7), G(N3))	0.8	0.7	0.0	3.3	Na(A(N1,N7),G(N3), P(1B1NB)
	BLYP	2.2	1.7	0.0	7.4	NaA(N7)	0.9	0.8	0.0	3.3	LiP(2B),Na(A(N7), P(1B1NB,2NB))
	BLYP-D3	7.0	6.0	0.3	26.3	KG(N3), RbA(N3,N7), CsA(N3,N7)	2.3	1.2	0.1	7.3	LiP(2B)
	BP86	3.4	3.1	0.1	13.3	Na(A(N1,N7), G(N3))	1.4	1.4	0.0	4.9	
	BP86-D3	6.8	7.0	0.1	27.5	K(A(N3), G(N3)), Rb/CsA(N1,N3,N7)	2.0	1.2	0.1	5.5	CsP(2B)
	PBE	2.3	1.8	0.0	6.9		0.9	0.6	0.0	2.5	LiP(2B), Na(A(N7), P(1B1NB,2NB))
	PBE-D3	2.4	2.6	0.0	11.9	K(A(N3), G(N3)), RbA(N3), CsA(N3)	0.8	0.8	0.0	4.2	Li(A(N3), P(1B1NB,2B))
	PBE-D4	2.2	2.4	0.0	11.9	KA(N3), RbA(N3), CsA(N3)	0.8	0.6	0.0	3.0	LiP(2B)
**Local LDA**	SVWN5	4.0	3.0	0.0	10.5		1.6	1.4	0.0	5.6	LiG(N7)

[a]Complexes resulting in an outlier in the boxplot statistics are listed according to the metal (Li^+, Na^+, K^+, Rb^+, or Cs^+) and nucleic acid component (A, C, T, G, U, or P), with the binding site indicated in parentheses. See Figure 2 for definitions of the complexes considered in present work and Figure 7 for the outliers.

Table S9. MPEs and MUEs for each DFT functional (with and without counterpoise corrections) relative to CCSD(T)/CBS reference values for group I metal complexes.[a]

**Metal**	**Functional**	**MPE**		**MUE**		**Absolute Diff.**[b]	
		w/o CP	w/ CP	w/o CP	w/ CP	MPE	MUE
Li^+	PBE-QIDH	1.0	0.7	0.8	0.4	0.3	0.4
	ωB97X-V	1.5	0.9	1.1	0.6	0.6	0.5
	O3LYP	1.7	1.0	1.2	0.6	0.7	0.6
Na^+	B2-PLYP-D3	0.9	1.1	0.6	0.4	0.2	0.2
	HSE06-D3	2.0	1.6	1.1	0.7	0.4	0.4
	PBE0-D3	1.8	1.5	1.0	0.6	0.3	0.4
	TPSS-D3	1.4	1.4	0.8	0.6	0.0	0.2
	PBE-D3	1.6	1.5	0.8	0.6	0.1	0.2
K^+	B2-PLYP	1.3	1.1	0.5	0.3	0.2	0.2
	ωB97X-V	1.4	1.1	0.6	0.3	0.3	0.3
	M06-2X	2.0	0.9	0.8	0.3	1.1	0.5
	MN15-L	1.8	1.3	0.5	0.4	0.5	0.1
Rb^+	mPW2-PLYP	2.5	1.0	0.9	0.4	1.5	0.5
	ωB97M-V	2.3	0.8	0.8	0.2	1.5	0.6
	MN15-L	2.9	1.8	0.9	0.4	1.1	0.5
Cs^+	B2-PLYP	1.8	1.0	0.6	0.2	0.8	0.4
	ωB97M-V	1.7	0.9	0.6	0.2	0.8	0.4
	ωB97X-V	1.7	1.0	0.6	0.2	0.7	0.4
	MN15-L	2.9	1.8	0.8	0.4	1.1	0.4
	mPW91	2.3	1.8	0.8	0.4	0.5	0.4
Group I	mPW2-PLYP	2.1	1.6	1.1	0.7	0.5	0.4
	ωB97M-V	1.7	1.1	0.8	0.4	0.6	0.4
	revTPSS	2.1	1.9	0.9	0.8	0.2	0.1
	TPSS	2.1	2.0	0.9	0.8	0.1	0.1

[a]See Figure 2 for the complexes considered in present work. [b]Absolute differences between counterpoise uncorrected and corrected for each functional are calculated for each metric.

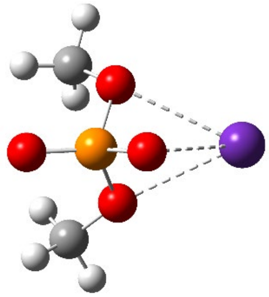

Figure S1. CCSD(T)/CBS//MP2/def2-TZVPP characterized P(2B) complex between each metal and the dimethylphosphate model, which includes an additional contact with the nonbridging oxygen for all metals except Li^{+}.

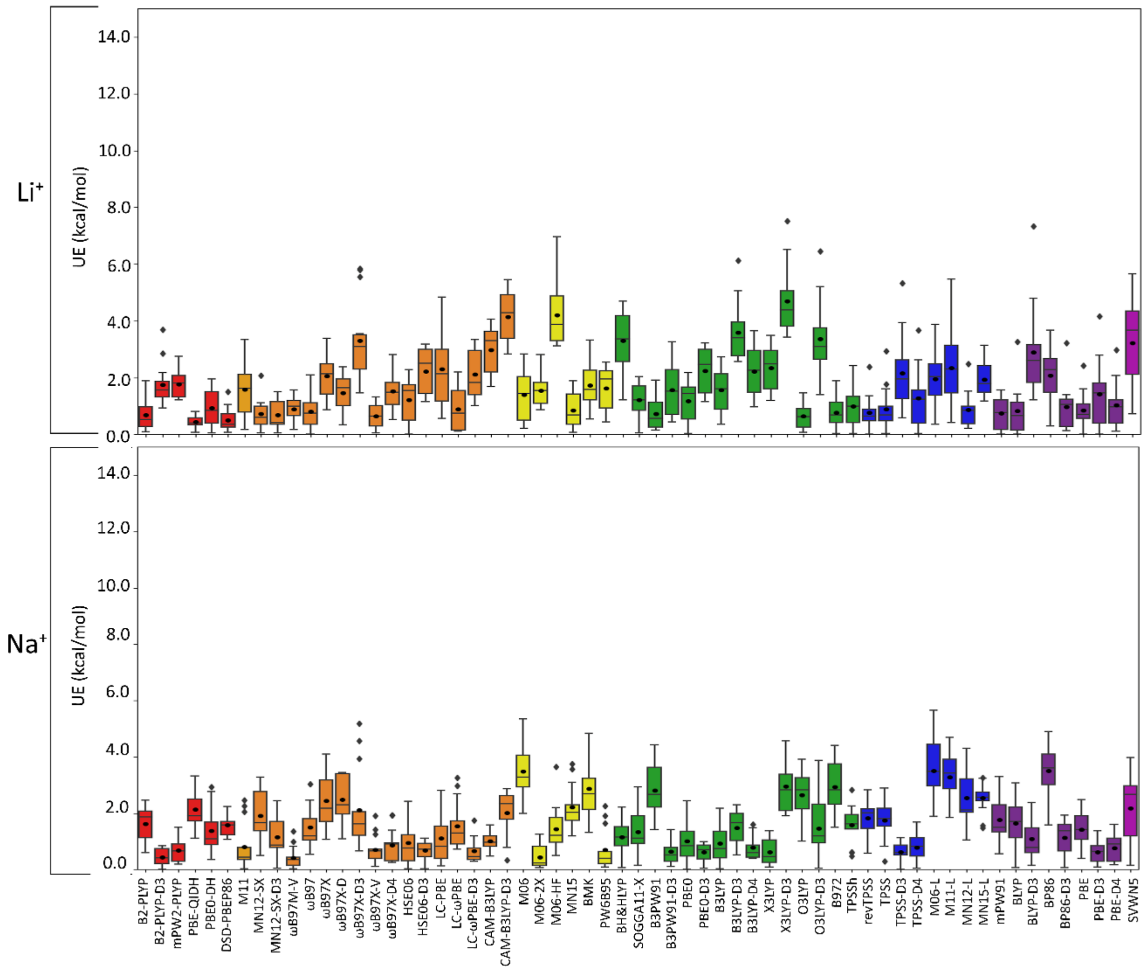

Figure S2. Boxplot plot statistics of the unsigned errors (kcal/mol) in Li^+ or Na^+–nucleic acid DFT binding energies relative to CCSD(T)/CBS reference values, with the functionals sorted according to double-hybrids (red), RSH (orange), GH meta-GGA (yellow), GH GGA (green), meta-GGA (blue), GGA (purple), and LDA (magenta).

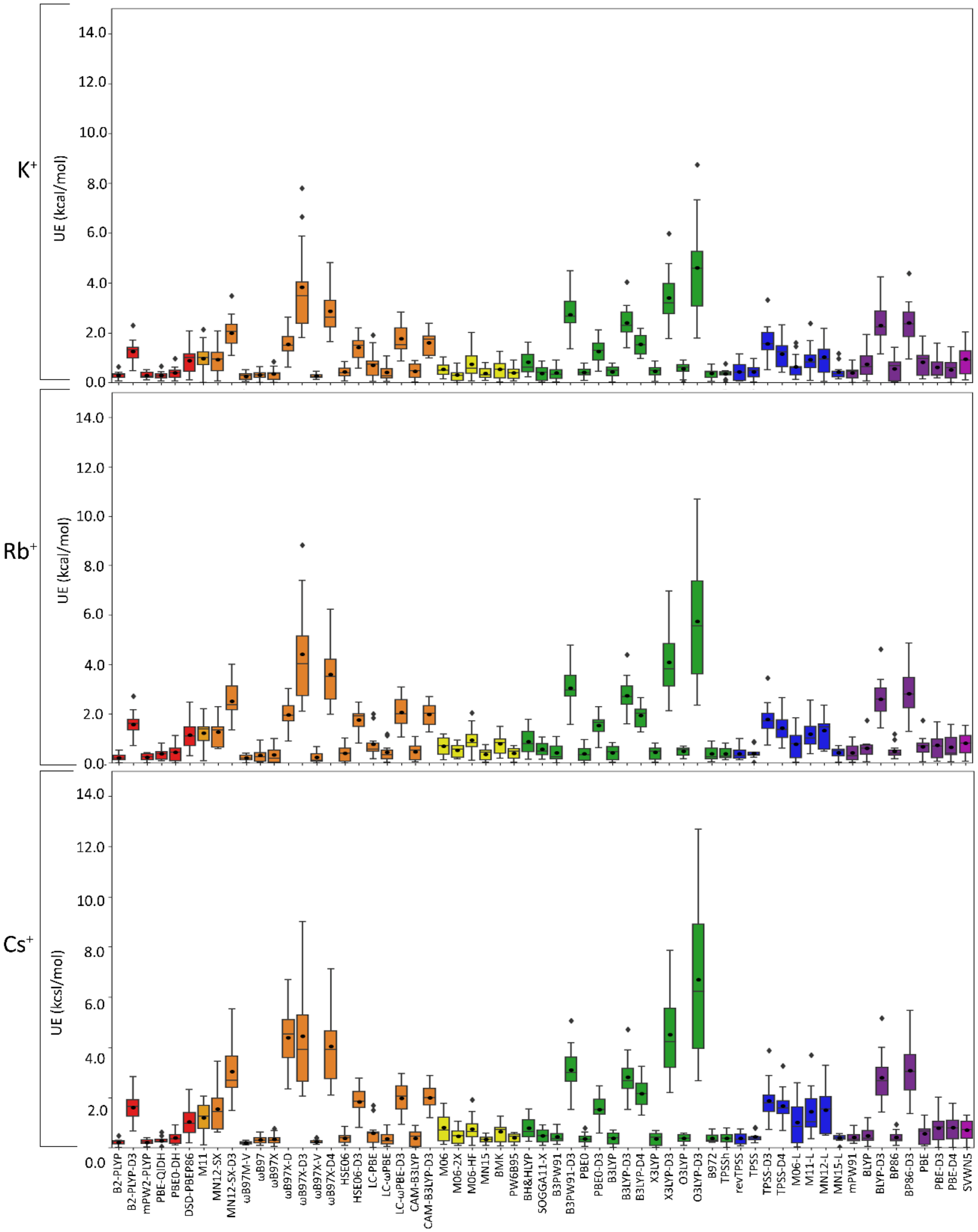

Figure S3. Boxplot plot statistics of the unsigned errors (kcal/mol) in K^+, Rb^+ or Cs^+–nucleic acid DFT binding energies relative to CCSD(T)/CBS reference values, with the functionals sorted according to double-hybrids (red), RSH (orange), GH meta-GGA (yellow), GH GGA (green), meta-GGA (blue), GGA (purple), and LDA (magenta).

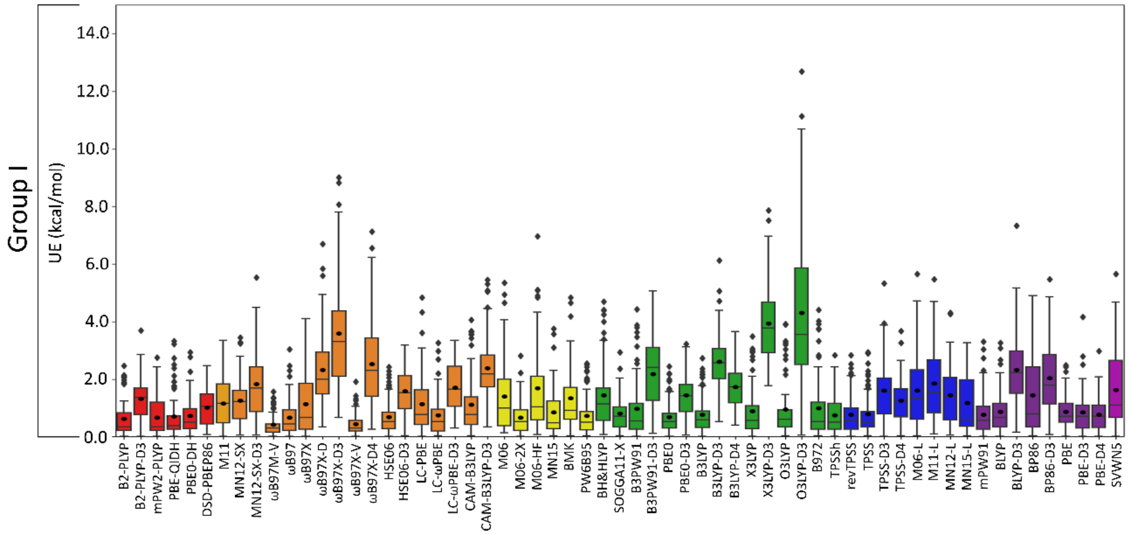

Figure S4. Boxplot plot statistics of the unsigned errors (kcal/mol) in group I metal–nucleic acid DFT binding energies relative to CCSD(T)/CBS reference values, with the functionals sorted according to double-hybrids (red), RSH (orange), GH meta-GGA (yellow), GH GGA (green), meta-GGA (blue), GGA (purple), and LDA (magenta).

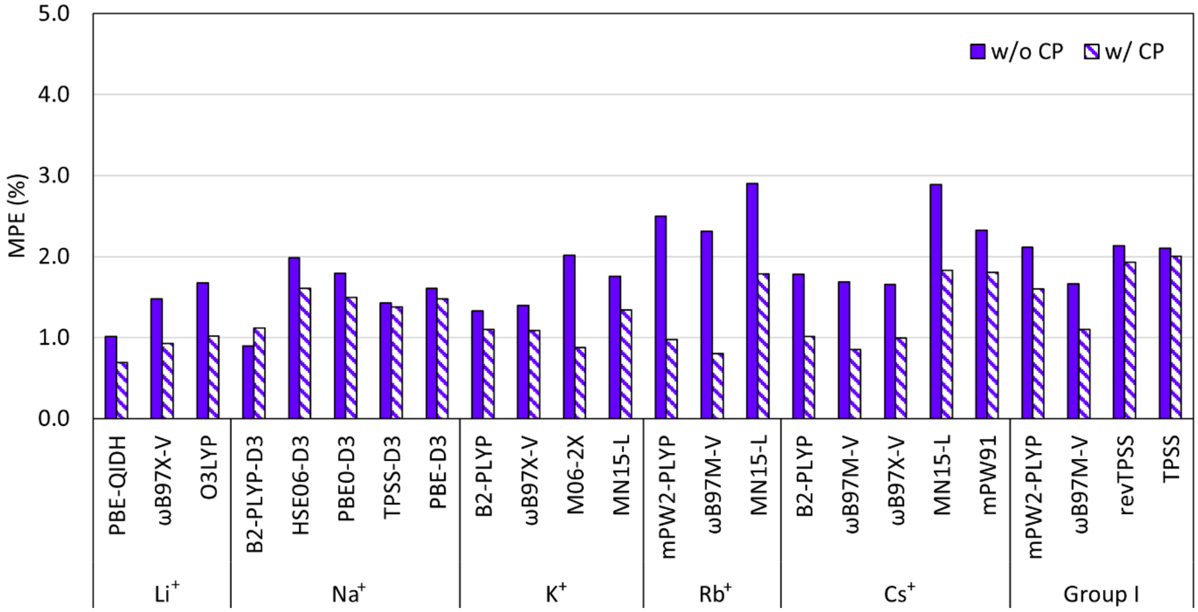

Figure S5: MPEs (%) in the uncorrected (solid) and counterpoise-corrected (striped) DFT binding energies relative to the CCSD(T)/CBS reference values for the top-performing functionals for each metal and over all group I.

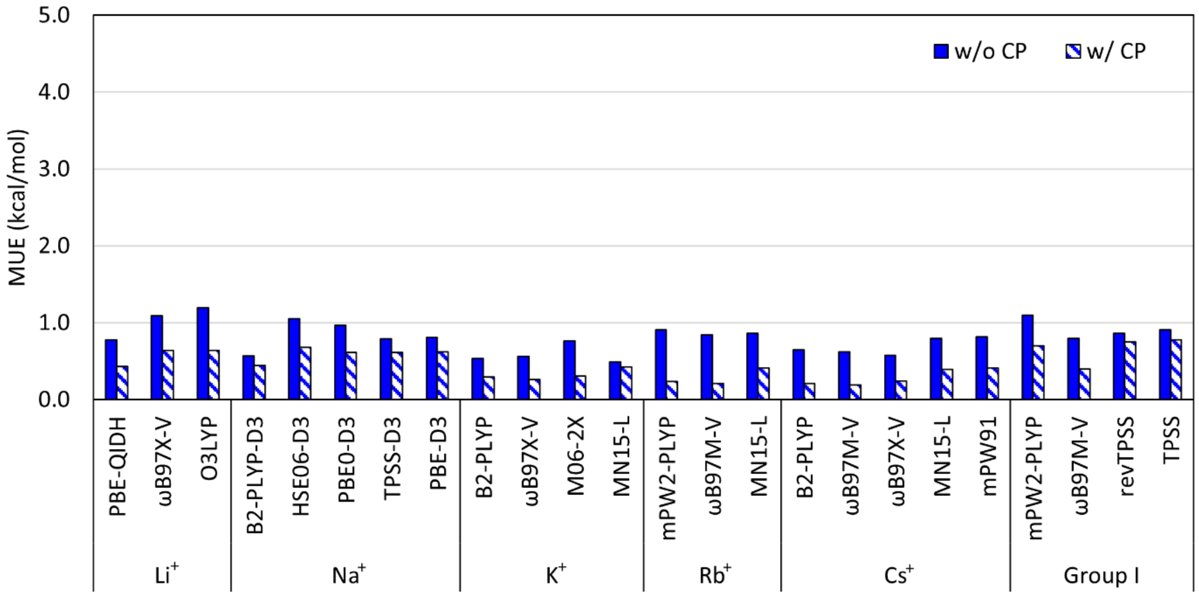

Figure S6: MUEs (kcal/mol) in the uncorrected (solid) and counterpoise-corrected (striped) DFT binding energies relative to the CCSD(T)/CBS reference values for the top-performing functionals for each metal and over all group I.
